# Repair spinal cord injury with a versatile anti-oxidant and neural regenerative nanoplatform

**DOI:** 10.1186/s12951-024-02610-5

**Published:** 2024-06-20

**Authors:** Heng Zhou, Ziwei Li, Shuili Jing, Ben Wang, Zhifei Ye, Wei Xiong, Yonghao Liu, Ye Liu, Chun Xu, Tushar Kumeria, Yan He, Qingsong Ye

**Affiliations:** 1 Center of Regenerative Medicine, Department of Stomatology, Renmin Hospital of Wuhan University, Wuhan, 430060 China; 2 The Second People’s Hospital of Linhai, Linhai, Zhejiang 317000 China; 3 School of Dentistry, The University of Sydney, Sydney, NSW 2006 Australia; 4 School of Materials Science and Engineering, University of New South Wales, Kensington, Sydney, NSW 2052 Australia; 5 Institute of Regenerative and Translational Medicine, Tianyou Hospital, Wuhan University of Science and Technology, Wuhan, 430064 Hubei China; 6 Department of Oral and Maxillofacial Surgery, Massachusetts General Hospital, Harvard Medical School, Boston, MA 02114 USA

**Keywords:** SeNPs, SeNPs@ZIF-8, Spinal cord injury, Reactive oxygen species, Neural regeneration

## Abstract

Spinal cord injury (SCI) often results in motor and sensory deficits, or even paralysis. Due to the role of the cascade reaction, the effect of excessive reactive oxygen species (ROS) in the early and middle stages of SCI severely damage neurons, and most antioxidants cannot consistently eliminate ROS at non-toxic doses, which leads to a huge compromise in antioxidant treatment of SCI. Selenium nanoparticles (SeNPs) have excellent ROS scavenging bioactivity, but the toxicity control problem limits the therapeutic window. Here, we propose a synergistic therapeutic strategy of SeNPs encapsulated by ZIF-8 (SeNPs@ZIF-8) to obtain synergistic ROS scavenging activity. Three different spatial structures of SeNPs@ZIF-8 were synthesized and coated with ferrostatin-1, a ferroptosis inhibitor (FSZ NPs), to achieve enhanced anti-oxidant and anti-ferroptosis activity without toxicity. FSZ NPs promoted the maintenance of mitochondrial homeostasis, thereby regulating the expression of inflammatory factors and promoting the polarization of macrophages into M2 phenotype. In addition, the FSZ NPs presented strong abilities to promote neuronal maturation and axon growth through activating the WNT4-dependent pathways, while prevented glial scar formation. The current study demonstrates the powerful and versatile bioactive functions of FSZ NPs for SCI treatment and offers inspiration for other neural injury diseases.

**Supplementary Information:**

The online version contains supplementary material available at 10.1186/s12951-024-02610-5.

## Introduction

Traumatic spinal cord injury (SCI) can result in temporary or permanent changes in motor and sensory function [1]. In traumatic SCI, the primary insult damages cells and triggers a complex secondary injury cascade that periodically leads to neuronal and glial cell death, ischemia, and inflammation [2]. This cascade is followed by changes in the tissue and structure of the spinal cord, including the formation of glial scars and cystic cavities, combined with poor regeneration of endogenous myelin and axon, meaning that the spinal cord has limited intrinsic recovery potential, so that SCI always leads to permanent neurological deficits [3].

Traumatic SCI is pathologically divided into primary and secondary injuries, and can be divided into acute (within 48 h), subacute (48 h to 14 days), intermediate (14 days to 6 months), and chronic (over 6 months) stages according to the period of progress [1]. The treatment of SCI faces different important challenges at different stages. During the acute to subacute phase, a large number of inflammatory cells congregate in the injured area, spread the inflammatory response and promote continuous apoptosis of neurons and oligodendrocytes [4]. During the progression of myelopathy from the dynamic metaphase to the chronic phase, the overwhelming cell death and degeneration in the acute phase of injury promote the formation of vesicles into cystic cavities, which combine to become a powerful barrier to axonal directed regeneration and a poor substrate for cell migration. There is a peri-pathological area around the sac lumen in which reactive astrocytes proliferate and become tightly intertwined to form an inhibitory mesh array. This glial scar strongly limits axonal regeneration (i.e. repair or regeneration of existing neural pathways, or development of new neural pathways) and anatomical plasticity by inhibiting neurite growth [5, 6].

Reactive oxygen species (ROS) is one of the main molecules that trigger changes in pathological processes [7]. ROS is a general term for a group of oxygen molecules derived from reductive oxidation reactions or electron excitation that have a close relationship with mitochondria, where the accumulation of ROS is associated with the loss of mitochondrial homeostasis, resulting in energy damage. Early after spinal cord injury, an increase in mitochondrial ROS cascade was observed in cells at the lesion site [8]. Inhibiting ROS production may protect damaged spinal cord tissue from mitochondrial oxidative stress, neuronal apoptosis, and axon death, as high levels of ROS impair the function of blood vessels to regulate inflammation, cell proliferation, and mitochondrial autophagy. Therefore, it is necessary to maintain mitochondrial homeostasis to control ROS production in cells after spinal cord injury [9]. The increase of ROS level interacts with inflammation level. Peri-vascular macrophages have been implicated in playing a key role in pathogenic neurovascular injury by increasing ROS levels [10]. After spinal cord injury, macrophage infiltration modulates neuroinflammation in the focal area. Compared to anti-inflammatory M2 macrophages, proinflammatory cytotoxic M1 phenotype situation cells play a leading role in the entire injury process, increasing blood-spinal cord barrier (BSCB) destruction and promoting endothelial cell apoptosis through neuroinflammation early in the injury [11]. In addition, mitochondrial DNA (mtDNA), which can exit mitochondria via BCL-2 antagonist/ killer 1 and BCL-2-associated X, apoptosis regulator pores or via the permeability transition pore complex, is a potent activator of cyclic GMP–AMP synthase, resulting in stimulator of interferon response cGAMP interactor 1 signaling and consequent synthesis of cytokines such as interferon-β1, interleukin 6 (IL-6) and tumor necrosis factor (TNF), which is account for the macrophage polarization [12].

Hence, ROS scavenging and inhibition of local apoptosis in SCI are the first challenge in the orderly treatment of SCI [13]. Rizwana et al. have synthesized a nanofiber scaffold based on polycaprolactone (PCL) loaded with the antioxidant graphene Oxide (GO) or Cerium Oxide nanase to remove excess ROS [14, 15]. Selenium nanoparticles (SeNPs) have attracted much attention because of their excellent ROS scavenging ability and anti-inflammatory effects [16, 17], and they can be used to remove excessive ROS deposition in animal models such as SCI, hepatic ischemia-reperfusion injury, and malignancies et al. [18–20]. Due to decreased selenium levels at the site of SCI injury, selenium supplementation is important for maintaining local homeostasis. However, SeNPs is a double-edged sword, because it has a narrow threshold between beneficial and toxic doses, leading to increasing concerns to their clinical application. In addition, excessive selenium can induce neuronal apoptosis [21, 22]. Therefore, there is an urgent need for a controlled selenium supplementation strategy that can combat ROS accumulation within a safe dose range.

**Scheme 1:**
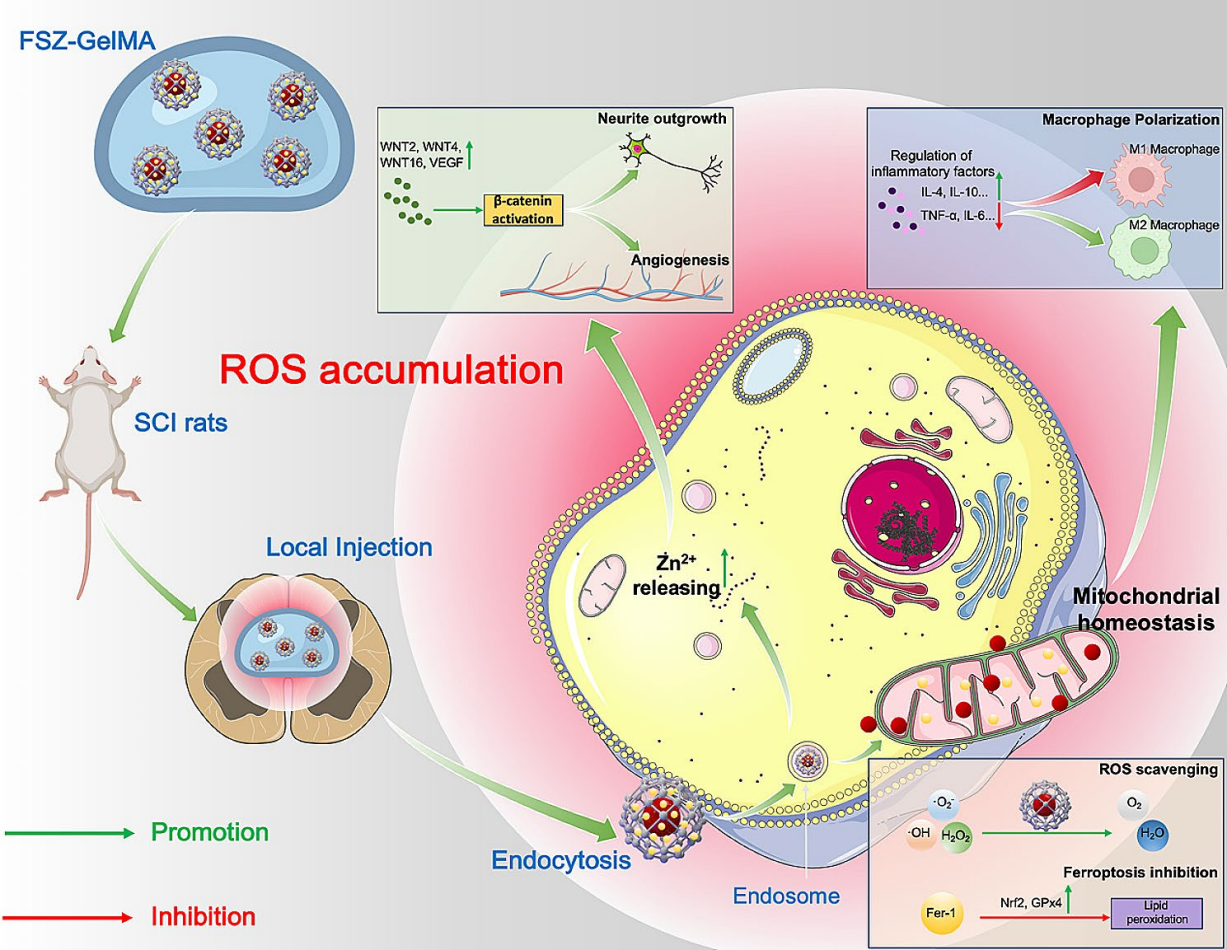
Schematic illustration of the FSZ nanoparticles loaded within GelMA hydrogel for the repair of acute spinal cord injury (SCI). SSe@ZIF-8 nanoparticles are coated with Ferrostatin 1 to obtained FSZ nanoparticles (FSZ NPs). FSZ NPs are suspended with in GelMA hydrogel and then injected locally into SCI rats

For the first time, we developed an innovative intensified antioxidant strategy to treat SCI through zeolitic imidazolate framework-8 (ZIF-8) capped selenium nanoparticles (SeNPs@ZIF-8) for enhanced ROS scavenging effect. Interestingly, we found that SeNPs based on different sizes, including small (2–10 nm), medium (15–30 nm) and large sizes (100–200 nm), formed different spatial structures from ZIF-8: alloy-like structures, core-shell structures and core-satellite structures. The 2-methylimidazole (2-MIM) of ZIF-8 nanoparticles can effectively enhance the antioxidant effect of the nanase, which may relatively reduce the toxicity of SeNPs [23]. Compared with SeNPs, the ROS scavenging capability of SeNP@ZIF-8 was enhanced. Recent evidences suggested that ferroptosis is one of the important targets for SCI treatment [24]. The sustainable inhibition of ferroptosis was performed by adding ferroptosis inhibitor ferrostatin 1 (Fer-1) to SeNPs@ZIF-8 (FSZ), which further increased the ability of the nanoplatform to resist lipid perioxidation [25]. This synergistic anti-oxidant nano-therapeutic strategy presented excellent ROS scavenging ability within a safe dose range to maintain mitochondrial homeostasis and prevent apoptosis of nerve cells. Mitochondrial maintenance further down-regulates the levels of inflammatory cytokines, which promotes the differentiation of macrophages into M2 phenotypes. In addition, our previous work indicated that ZIF-8 nanomaterials can promote the neural differentiation and pro-angiogenic abilities of mesenchymal stem cells (MSCs) and increase their axon length by releasing Zn^2+^ [26]. This suggests that FSZ may provide additional nerve regeneration and angiogenesis capacity to response the cascade of SCI (Scheme 1). The present work has presented a novel antioxidant treatment strategy to avoid toxicity of SeNPs, which provides not only a promising treatment for SCI but also an inspiration for other acute neural injury diseases, such as stroke, traumatic brain injury and peripheral nerve injury.

## Materials and methods

### Chemicals and materials

All chemicals were obtained from commercial suppliers and used as received without further purification. Zinc nitrate hexahydrate (Zn(NO_3_)_2_·6H_2_O), selenocystine, methanol, 30% H_2_O_2_, and tert-Butyl hydroperoxide (TBHP) were purchased from Macklin (Shanghai, China). 2-MIM, polyvinylpyrrolidone (PVP, Mw = 40,000), dopamine hydrochloride, Sodium selenite pentahydrate (Na_2_SeO_3_·5H_2_O), NaOH were purchased from Aladdin (Shanghai, China). 3,3’,5,5’-tetramethyl benzidine (TMB), 2,2′-Azinobis-(3-ethylbenzthiazoline-6-sulphonate) (ABTS), Cell Cycle and Apoptosis Analysis Kit, Annexin V-FITC Apoptosis Detection Kit, 2′,7′-Dichlorofluorescein diacetate (DCFH-DA), Calcein AM, BCA Protein Assay Kit and crystal violet dye were purchased from Beyotime Biotechnology (Shanghai, China). Cell staining buffer, Human VEGF-a, TNF-α, IL-6 and IL-4 ELISA Kit were purchased from Elabscience (Wuhan, China). Chlorpromazine (CPZ), Methyl-β-cyclodextrin (MβCD), chloroquine (CHL), LY294002, MitoSOX Red, Cell Counting Kit-8 (CCK-8), radioimmunoprecipitation assay (RIPA) lysis and cocktail are purchased from MedChemExpress (USA). PC12 cell line (Undifferentiated) and Human Umbilical Vein Endothelial Cells (HUVECs) were supplied by Procell Life Science&Technology (wuhan, China). Sodium dodecyl sulfate-polyacrylamide gels (SDS-PAGE) are purchased from Epizyme Biotech (Shanghai, China). Cell culture plate, Matrigel and transwell chambers (8.0 μm pore size, 24-well) were purchased from Corning (USA). Hematoxylin and Eosin (H&E), Nissl Stain Kit, and TUNEL Apoptosis Assay Kit were purchased from Solarbio Science & Technology (Beijing, China). N- (6-Methoxy-8-quinolyl)-p-toluenesulfonamide) (TSQ) was purchased from enzo life science (USA). Roswell Park Memorial Institute 1640 medium (RPMI-1640), dulbecco’s modified eagle medium (DMEM), fetal bovine serum (FBS), Horse Serum (HS), Phosphate-buffered saline (PBS), and polyvinylidene fluoride (PVDF) membrane were purchased from Thermo Fisher Scientific (USA). The GelMA hydrogel is purchased from QiYue Biology (Xian, Shanxi, China). Endothelial cell medium (ECM) was purchased from Sigma-Aldrich. PE-Cy7 Rat Anti-Mouse CD86, Alexa Fluor 647 Rat Anti-Mouse CD206, Fixation/Permeablization Kit were purchased from BD Pharmingen (USA).

### Synthesis of nanomaterials

#### Synthesis of ZIF-8

5 mL of 62.3 mM 2-MIM (25.6 mg) methanol solution AND 5 mL of 16.9 mM Zn(NO_3_)_2_·6H_2_O (25.2 mg) methanol solution were mixed and stirred for 30 min at 37 ℃. The products were centrifuged at 12,000 rpm for 5 min and washed with methanol three times.

#### Synthesis of SeNPs

Small, medium and large size nano-selenium was prepared by mixing 1 ml 25 mM sodium selenite with 4 ml 25 mM glutathione (containing 200, 20 and 2 mg BSA), respectively. The pH of the solution was adjusted to 7.2 with 1.0 M sodium hydroxide to form red element Se and glutathione oxide (GSSG). The red solution was dialyzed on double distilled water for 96 h, and the water was changed every 24 h to separate GSSG and Nano-Se. The final solution containing nano-selenium and bovine serum albumin was freeze-dried and stored at room temperature. Transmission electron microscopy (TEM) revealed that the size of red element Se is 5–15 nm (small size, designed as SSSe), 20–60 nm(medium size, designed as SSe) and 80–200 nm(large size, designed as Se).

#### Synthesis of Se@ZIF-8

10 mg SeNPs and 500 mg PVP were added to 10 mL methanol and stirred for 2 h at room temperature. The PVP-SSe was collected by centrifugation at 100,000 g for 60 min. The obtained PVP-SSSe was added to 5 mL of 62.3 mM 2-MIM (25.6 mg) methanol solution and stirred for 15 min at 37 ℃. Subsequently, 5 mL of 16.9 mM Zn(NO_3_)_2_·6H_2_O (25.2 mg) methanol solution was added to this mixed solution and stirred for another 30 min at 37 ℃. The products were centrifuged at 12,000 rpm for 5 min and washed with methanol three times.

#### Synthesis of Fer-1@SSe@ZIF-8 (FSZ)

40 mg SSe@ZIF-8 and 26.2 mg Fer-1 were added to 1 mL DMSO and the mixture stirred continuously at room temperature and dark for 24 h. The products were centrifuged at 12,000 rpm for 5 min and washed with DMSO three times. The resultants were dried at ambient temperature in a vacuum oven overnight.

### Nanomaterials characterization

TEM and energy spectra analysis was observed with JEM-2100 and JEM-F200 TEM (JEOL, Japan). X-ray diffraction (XRD) data was obtained with a XPert Pro diffractometer (Panalytical, Holland).

### Cell culture

PC12 cells were incubated in RPMI-1640 medium with 15% HS and 5% FBS at 37 °C in a 5% CO_2_ environment. The culture medium was changed every 3 days. HUVECs were incubated with ECM at 37 °C in a 5% CO_2_ environment. The culture medium was changed every 1 days. RAW264.7 cells were incubated with DMEM containing 10% FBS at 37 °C in a 5% CO_2_ environment. The culture medium was changed every 1 days. MSCs were extracted and identified as before [27]. MSCs were incubated with α-MEM containing 10% FBS at 37 °C in a 5% CO_2_ environment. The culture medium was changed every 3 days.

### Intracellular uptake of nanoparticles

To visualize SSe@ZIF-8, coumarin 6 (C6) is mixed with 2-MIM solution along with PVP-Se before adding Zn(NO_3_)_2_·6H_2_O. After similar steps, 20 μg/ml C6-labeled Se@ZIF-8 (C6-SSe@ZIF-8) is obtained. PC12 cells (5 × 10^4^ cells per well) were seeded in a 12-well plate and attached overnight. The cells were stained with Hoechst 33,342 (blue) for nucleus and LysoTracker (red) for lysosome, and C6-SSe@ZIF-8 (green) was added. At 0, 1, 2, and 4 h, the medium was removed and cleaned 3 times with pre-cooled PBS. real-time living cell imaging were captured by an inverted fluorescence microscope (IX71, OLYMPUS). To further explore the intracellular uptake of SSe@ZIF-8, endocytotic inhibitor CPZ (10 μg/mL), MβCD(5 mM), CHL(20 μM), and LY294002 (100 μM) was used to pretreat cells for 1 h.

### TEM analysis for cellular localization of nanoparticles

PC12 cells (5 × 10^4^ cells per well) were seeded in a 12-well plate and incubated with 20 μg/mL SSe@ZIF-8 for after adhesion. After 6 h, the cells were collected and washed. After fixation and gradient dehydration, the samples were coated with epoxy resin and kept overnight in an oven at 60 °C. Ultrathin sections were prepared and stained with uranyl acetate and lead citrate for 15 min, and observed under TEM.

### PH-release drug release

Fer-1 was labeled with Cy5.5 and Cy5.5-Fer-1@SSe@ZIF-8 was prepared for detection of drug release properties. SSe@ZIF-8 (20 μg/mL) was dispersed in 10mL solution of different pH (pH = 5.5, 6.5, and 7.4, respectively) at 37 °C. At each time point, 1mL of solution was taken and centrifuged at 12,000 rpm for 5 min, and the absorbance of supernatant was measured at 450 nm by a microplate reader (PerkinElmer, EnSight).

### TMB and ABTS free radical scavenging assay

To assess antioxidant activity of nanoparticles, the TMB and ABTS scavenging assays were used. According to the instruments, TMB (80 mM) was added into 1 mL of solution with 100 μM H_2_O_2_. The absorbance changes of TMB at 654 nm were monitored. The ABTS•+ was mixed with different concentrations of nanoparticles. The absorbance of ABTS at 734 nm was examined using a a microplate reader (PerkinElmer, EnSight),

### Mitochondrial ROS detection

Mito-Tracker Red probes are used to detect mitochondrial superoxide production. PC12 cells (5 × 10^4^ cells per well) were seeded in a 35 mm confocal dish and attached. After 24 h stimulation with 200 μM H_2_O_2_, the cells were incubated with Mito-Tracker Red for 10 min at 37 °C and stained with Hoechst 33,342. Intracellular fluorescence was observed by a confocal laser scanning microscopy (CLSM, FV1200, OLYMPUS).

### Mitochondrial membrane potential detection

PC12 cells were treated with TBHP and FSZ NPs for 24 h. After the culture-medium was removed, the cells were gently washed with PBS, and 1 ml JC-1 staining solution (Beyotime, Shanghai, China) was added to each well and incubated ina cell incubator at 37 °C for 15 min. Wash gently with washing buffer 2 times and add fresh medium. Fluorescence microscopy (Leica DM3000 LED) measures fluorescence intensity.

### Cell viability assay

The cytotoxicity of SSe, ZIF-8 and SSe@ZIF-8 was measured using CCK-8. PC12 cells (5 × 10^3^ cells per well) were seeded in a 96-well plate and adhered overnight. Different concentrations of SSe, ZIF-8 and SSe@ZIF-8 were added to the medium and the cells were cultured for 24 h. After incubation with freshly prepared CCK-8 solution at 37 ℃ for 2 h, the absorbance was measured at 450 nm by a microplate reader (PerkinElmer, EnSight).

### Cell cycle analysis

The effect and recovery ability of SSe@ZIF-8 on PC12 cell cycle were tested by flow cytometry. With or without TBHP (100 μM) treatment, cells were incubated with different concentrations of SSe@ZIF-8 for 24 h. The cells were fixed overnight with 75% ethanol solution at 4 °C, incubated with propidiumiodide (PI) and ribonuclease (RNase) at 37℃ for 30 min, and then analyzed by flow cytometry (CytoFlex, Beckman Coulter).

### Intracellular ROS detection

Intracellular ROS levels were evaluated using DCFH-DA probes. MSCs with multiple differentiation potential (5 × 10^4^ cells per well) were seeded in a 12-well plate and attached. SSe (20 μg/mL), ZIF-8 (20 μg/mL), Fer-1 (0.1 μM), SSe@ZIF-8 (40 μg/mL) and FSZ (40 μg/mL) were added according to the groups, and the cells were incubated with TBHP (100 μM) for 24 h. Then cells were incubated DCFH-DA (10 μM) at 37 °C for 30 min and observed by a CLSM (FV1200, OLYMPUS). The ratio of average fluorescence intensity of ROS-stained cells was measured in each field by ImageJ.

### Cell apoptosis analysis

Apoptosis was evaluated by Annexin V/PI staining of flow cytometry. Under the stimulation of TBHP (100 μM), PC-12 cells were incubated with SSe (20 μg/mL), ZIF-8 (20 μg/mL), Fer-1 (0.1 μM), SSe@ZIF-8 (40 μg/mL), and FSZ (40 μg/mL) according to the groups. After 24 h, cells were stained with Annexin V FITC and (PI), and analyzed using a flow cytometer (CytoFlex, Beckman Coulter). In post-analysis, cell apoptosis was determined by the following rules (active cells: annexin V‐ and PI‐; early apoptotic cells: annexin V + and PI‐; late apoptotic cells: annexin V+ and PI+; dead cells: annexin V‐ and PI+).

### Measurement of neurite growth

The direct effects of nanomaterials on synaptic growth in PC12 cells, a classic model for neuron growth, differentiation, neurotransmitter release, and neurodegenerative diseases, and the correlation with dose or time were explored. PC12 cells were treated with different concentrations (0, 5, 10, 20, 40, and 80 μg/mL) of SSe@ZIF-8 and cultured in ordinary medium. After 6 days, the cells were observed under a optical microscope. Next, PC12 cells with the same treatment were cultured in DMEM with 10 ng/mL NGF. At 2, 4, and 6 d, the PC12 cells were stained with Calcein AM (1 μM) for 30 min at 37 °C in the dark and then replaced with fresh medium for another 30 min. The morphology of PC12 cells was observed and the number and length of neurites were analyzed under a fluorescence microscope (BX53, OLYMPUS). In growth ratio analysis, differentiated PC12 cells are divided into L0-L6 according to the ratio of their longest neurite length to the diameter of the cell body. For example, L0 is defined as cells whose longest neurite length is shorter than the diameter of the cell body; L1 is defined as cells whose longest neurite length is between the original and twice the diameter of the cell body.

### Transcriptome analysis

The detailed steps were shown in our previous study. In short, 1 × 10^7^ MSCs were incubated in B-27 Plus Neuronal Culture System (20 ng/ml EGF and 20 ng/ml bFGF), treated with 40 μg/mL FSZ for 6 days. Total RNA was extracted and qualified. Strand RNA sequencing libraries were prepared. PCR products corresponding to 200–500 bps were enriched, quantified and sequenced. RNA-Seq data analysis Raw sequencing data were first filtered by Trimmomatic (version 0.36). Limma packets were used to identify genes that were differentially expressed between groups. A *p*-value cutoff of 0.05 and a fold-change cutoff of 1.5 were used to judge the statistical significance of differenced gene expressions (DEGs). Gene ontology (GO) analysis and Kyoto encyclopedia of genes and genomes (KEGG) enrichment analysis for DEGs were both implemented by DAVID (https://david.ncifcrf.gov) with a *p*-value cutoff of 0.05 to judge statistically significant enrichment.

### Western blot analysis

After transfection with WNT4-siRNA or control siRNA (the sequences was shown in Table S1), PC12 cells were treated with a series of concentrations of FSZ and differentiated for 6 days. Raw264.7 cells are stimulated by LPS (1 mg/mL) and incubated with Fer-1 (0.1 μM), SSe@ZIF-8 (40 μg/mL), and FSZ (40 μg/mL) for 24 h. Proteins were extracted with RIPA lysis containing cocktail and their concentrations were detected by BCA. These protein samples were separated by SDS-PAGE electrophoresis and transferred to PVDF membranes. After blocking, the membranes were incubated overnight at 4℃ with primary antibody including anti-Nestin (Santa Cruz, sc-23,927 trail size, 1:200), anti-TUJ1 (abcam, ab105389, 1:5000), anti-VEGF-a (abcam, ab46154, 1:5000), anti-GAPDH (Servicebio, GB15002, 1:10,000), anti-iNOS (CST, 10,320, 1:2000), anti-Arg1 (abcam, ab239731, 1:5000), anti-TNFα (CST, 11,948, 1:10,000), anti-IL10 (Cell Signaling Technology, 12,163, 1:5000), Axin2 (Proteintech, 20540-1-AP, 1:2000), Active β-catenin (Cell Signaling Technology, 8814 S, 1:2000), β-catenin (Servicebio, GB11015, 1:1000), GSK3β (abcam, ab32391, 1:2500), p-GSK3β (Abcam, ab75814, 1:2500), anti-JNK1 (Cell Signaling Technology, 9252P, 1:500), anti-p-JNK1 (Cell Signaling Technology, 9255 S, 1:500), anti-p38 (Cell Signaling Technology, 8690P, 1:500), and anti-p-p38 (Cell Signaling Technology, 4511 S, 1:500), primary antibody. The next day, the bands were incubated with the corresponding secondary antibody and scanned with gel imaging system (ChemiDoc, BIO-RAD).

### Enzyme-linked immunosorbent assay (ELISA)

PC12 cells were incubated with a series of concentrations of FSZ NPs, respectively. The supernatants were collected and subjected to VEGF-a/IL-4/IL-6/TNF-α ELISA assay according to the specification.

### Transwell migration assay

PC12 cells were cultured with ECM medium with different concentrations of SSe@ZIF-8 for 24 h. HUVECs (1 × 10^4^ cells per well) were resuspended in 100 μL ECM from PC12 cells (with different concentrations of SSe@ZIF-8) and then seed in the upper chambers of the 24-well transwell nest. Meanwhile, medium containing FBS was added into the lower chamber. After incubation at 37 °C for 24 h, the cells on the surface of the membrane were washed with PBS, fixed with 4% paraformaldehyde for 15 min and stained with 0.1% crystal violet for 30 min at room temperature. Cells on the upper surface of the transwell membrane were scraped out. After that, cells were photographed and five fields were randomly selected to count the number of migrated cells on the back surface.

### Cell homing detection

PC12 cells (2 × 10^4^ cells per well) were seeded in the upper chambers of the transwell nest, while different concentration of FSZ-GelMA hydorgel were fixed in bottom of the upper chambers with visible light source. The cell viability test was performed by CCK-8 assay.

### Tube formation assay

The matrigel-coated plate was obtained by adding 60 μL matrigel (1:1) to each hole of the 96-well plate and leaving it at room temperature for 15 min and 37 °C for 60 min. HUVECs (1 × 10^4^ cells per well) were seeded in each matrigel-coated well and incubated with ECM containing different concentrations (0, 5, 10, 20, 40, and 80 μg/mL) of SSe@ZIF-8 for 6 h. The tubular structures were observed under inverted fluorescence microscope (IX71, OLYMPUS). The tube length and the number of nodes and meshes were determined by the ImageJ software (Media Cybernetics).

### Flow cytometry

Except the control group, RAW264.7 cells were stimulated with LPS (1 mg/mL) and the cells in experimental group were treated with Fer-1 (0.1 μM), SSe@ZIF-8 (40 μg/mL), and Fer-1@SSe@ZZIF-8 (40 μg/mL), respectively. After incubation for 24 h, single-cell suspension was blocked with Fc Block and stained with CD86 at 4 °C for 30 min. Then samples were stained with CD206 at 4 °C for 30 min after fixation and permeabilization. A flow cytometer (CytoFlex, Beckman Coulter) is used to analyze the ratio of M1/M2 cell polarization.

### Animals and ethics statement

36 female Sprague-Dawley rats (4 weeks old) were purchased from the Experimental Animal Center of Three Gorges University (Yichang, Hubei, China) and adaptively fed at room temperature and adequate diet for a week. All surgical procedures were approved by the Experimental Animal Ethics Committee of Renmin Hospital of Wuhan University (20220704B).

### Surgical procedure and treatment

To establish the SCI model, rats were anesthetized by intraperitoneal injection of 3% phenobarbital (0.1 mL/100 g) and the skin was disinfected. Then a longitudinal incision is made, and the T10 laminectomy was conducted to expose the spinal cord. Except the sham operation group, the spinal cord was compressed by a vascular clamp with a closing force of 50 g for 15 s, and the tail of rats swung from side to side and then suddenly drooped, indicating the model is successful. The muscle, fascia and skin were sutured layer by layer. After the establishment of the model, the sensory and motor functions of the hind limbs of rats were lost with a Basso, Beattie, and Bresnahan (BBB) score of < 2. For 3 days after surgery, penicillin was injected intramuscularly once a day. The bladder was manually emptied twice daily until the urinary function was restored.

All rats were randomly divided into 6 groups (*n* = 6): (1) sham operation group, (2) SCI model group, (3) GelMA (the pure GelMA hydrogel), (4) Fer-1 (GelMA with 1 μM Fer-1), (5) SSe@ZIF-8 (GelMA with 40 μg/mL SSe@ZIF-8), (6) Fer-1@ZIF-8(GelMA with 40 μg/mL FSZ). Rats in the sham operation group only received laminectomy without spinal cord compression. At 24 h post injury, 10 μL of corresponding GelMA hydrogel was injected into the spinal cord injury site by stereotaxic instrument and microinjection pump, and solidified with a light curing lamp for 10 s. The SCI model group was treated with an equal volume of saline as the control.

### Behavioral tests

The spinal cord function in rats was evaluated by video recordings, Basso, Beattie & Bresnahan (BBB) Locomotor Rating Scale, oblique plate test, and footprint analysis on 1, 7, 14, 21 and 28 d post-SCI. The rats were asked to walk along the wall, and videos were taken. height from the ground, less foot error, spasm duration and forward distance were analyzed. BBB scores were performed by three trained observers who were blinded to the experimental design. For footprint analysis, the rats were asked to run in a narrow dark track (10 cm × 100 cm) lined with paper. The red ink on the sole of the foot could record the position of footprints when rats touched. For the slope test, the rats were placed on an angle-adjustable inclined plate and the angle between the inclined plate and the desktop was slowly increased. When the rat could only stay in the original position for 5 s, the angle was recorded.

### Blood routine and biochemical tests

On Day 28 after SCI, Anticoagulant blood for rat tail was taken for routine blood test provided by Servicebio company.

### H&E staining

On Day 28 after SCI, the rats were given saline and 4% PFA for cardiac perfusion. The spinal cord from T9-T11 was carefully separated and fixed with 4% paraformaldehyde. The tissues were embedded in paraffin after dehydration and made into paraffin sections (5 μM thickness). The sections were baked at 60 °C for 1 h and then dewaxed with xylene and gradient ethanol. The sections were stained with H & E Stain Kit and photographed under an optical microscope.

The heart, liver, spleen, lung and kidney of the rats were also extracted and stained.

### Nissl staining

Slices were incubated with Nissl staining solution at 37 °C for 3 min, and then washed with distilled water and 95% ethanol. After dehydration, transparency and sealing, images were obtained under an optical microscope.

### Immunofluorescence staining

The tissue sections were incubated with 1% BSA for 30 min after antigen repair. Subsequently, the samples were incubated with anti-iNOS (CST, 10,320, 1:1000), anti-Arg1 (abcam, ab239731, 1:2000), anti-Tuj1 (CST, 5568, 1:2000), anti-GFAP (CAT, 3670, 1:1000), anti-Nrf2 (CST, 12,721, 1:1000), and anti-c-cap3 (santa cruz, sc-56,053, 1:100) primary antibody, and then incubated with corresponding FITC-labeled goat anti-mouse and Cy3-labeled goat anti-rabbit secondary antibody for 1 h at RT in the dark. Finally, they were stained with DAPI, followed by sealing with anti-fluorescence quenching sealing tablets. The fluorescence distribution of these cells was observed by a fluorescence microscope (BX53, OLYMPUS) and analyzed by the ImageJ software.

Raw264.7 cell samples were obtained by the same treatment as those for FCM. Cells were fixed with 4% paraformaldehyde and permeabilized with 0.2% Triton X-100. The next protocol was similar to the above.

### Immunohistochemical staining

The sections were blocked with 1% bovine serum albumin (BSA) at RT for 60 min, and incubated with anti-CD31 (abcam, ab28364) primary antibody overnight at 4 °C . Afterwards, the samples were incubated with the secondary antibody at RT for 1 h and stained with DAPI. Images were collected with a fluorescence microscope (BX53, OLYMPUS) and analyzed using ImageJ.

### TUNEL staining

The sections were incubated with the TUNEL reaction mixture for 60 min at 37 °C in the dark, followed by stained with DAPI for 5 min at RT. Images were obtained with a fluorescence microscope (BX53, OLYMPUS) and analyzed using ImageJ. The apoptotic cell proportion was calculated as the number of TUNEL-positive cells / total number of cells.

### TSQ staining

TSQ is a fluorophore that binds intracellular Zn ion [28]. The sections were immersed with 4.5 μM TSQ in 140 mM sodium barbital and 140 mM sodium acetate buffer (pH 10) for 90 s. Images were obtained with a fluorescence microscope (BX53, OLYMPUS) and analyzed using ImageJ (Version 1.53a for Mac).

### Statistical analysis

All experiments in this paper were repeated three times. Data were analyzed using GraphPad Prism (Version 9.0 for Mac) were expressed as mean ± standard deviation (Mean ± SD). Statistical analysis of experimental data was performed using one-way ANOVA followed with Bonferroni’s post-hoc test or paired t-test (*P < 0.05, **, ##*P* < 0.01, and ***P < 0.001).

## Results

### Size-dependent synthesis strategy of ZIF-8 encapsulated SeNPs

**Fig. 1:**
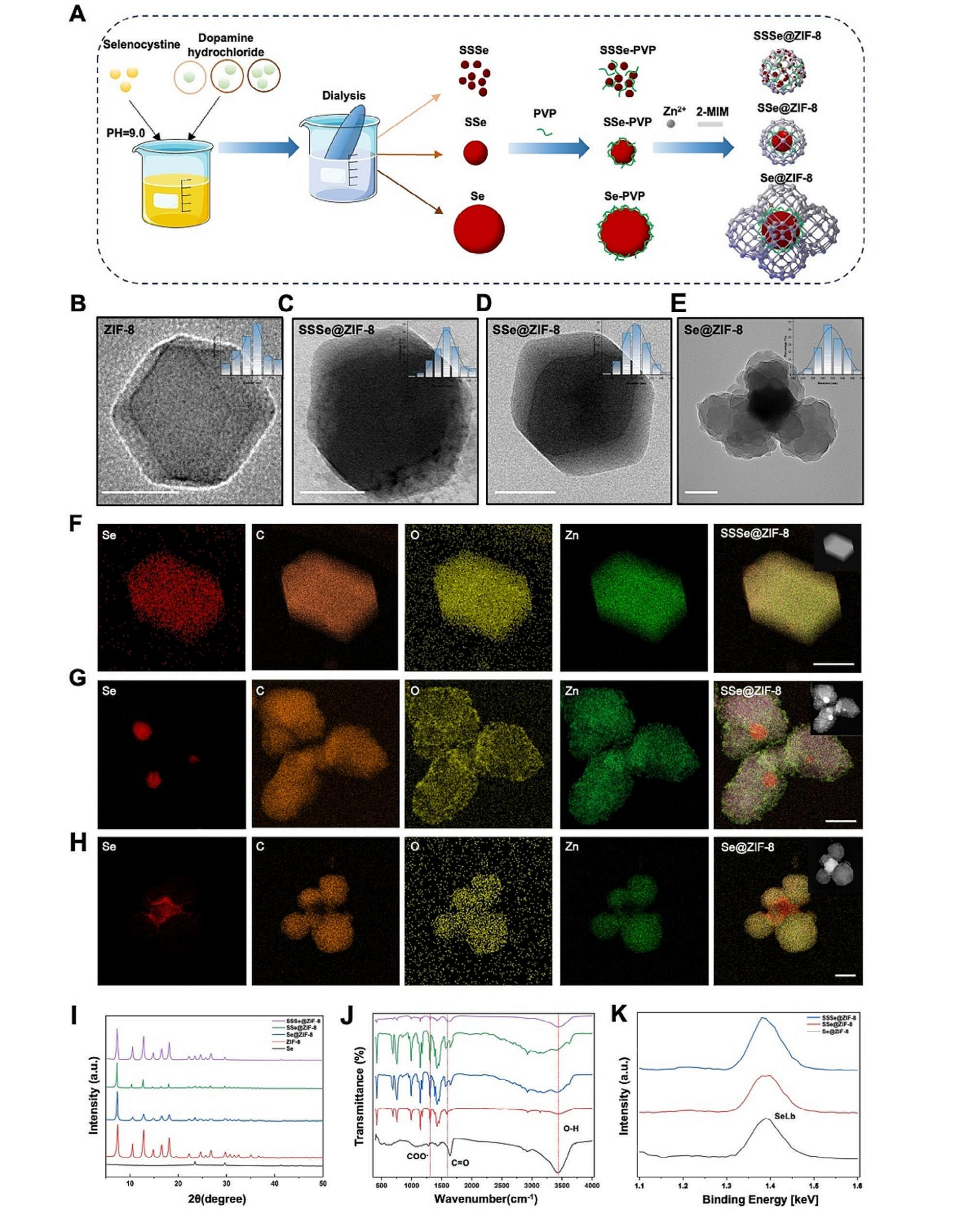
Structural characterization of SeNPs@ZIF-8 nanomaterials. **A**) Schematic illustration of the assembly procedure of FSZ NPs. Selenium nanoparticles with different sizes were synthesized and assembled with ZIF-8 to generate three novel composite nanomaterials. After the anti-ROS activity detection, SSe@ZIF-8 were selected to combine with Fer-1 to form FSZ NPs. **B**-**E**) TEM images and size distribution of ZIF-8, SSSe@ZIF-8, SSe@ZIF-8, and Se@ZIF-8. Scale bar: 100 nm. **F**-**H**) TEM-EDX elemental mapping and HAADF-STEM images of SSSe@ZIF-8, SSe@ZIF-8, and Se@ZIF-8. Scale bar: 50 nm. **I**) XRD pattern, **J**) FT-IR spectra, **K**) energy spectrum analysis of SSSe@ZIF-8, SSe@ZIF-8, and Se@ZIF-8

SeNPs could be controlled in size ranging from 2 to 200 nm by reducing selenite in an environment containing PVP, which attaches to Se atoms and stops their aggregation [29]. In this study, three different sizes of SeNPs were synthesized. On this basis, ZIF-8 was reasonably modified into a series of novel nanomaterials using three encapsulation strategies (Fig. 1A) in pursuit for enhancing antioxidant activity and pro-neural differentiation function in non-toxic dose. Different from conventional metal-core bimetallic MOF materials, this synthesis strategy was based on nonmetallic SeNPs. As shown in Fig. 1B, the ZIF-8 nanoparticles exhibit a typical hexagonal structure (70.2 ± 6.7 nm). Interestingly, based on the size of the SeNPs, these MOF composites with non-metallic core exhibit structural characteristics similar to those of bimetallic nanocrystals.1) The smallest-sized selenium nanoparticles (2.3 ± 0.3 nm, Fig. S1A) were called Small-small Selenium (SSSe), which combined with ZIF-8 to form the alloy-structured SSSe@ZIF-8 (97.4 ± 7.0 nm; Fig. 1C). 2) The medium-sized selenium nanoparticles (21.6 ± 2.1 nm, Fig. S1B) were called Small Selenium (SSe), which were encapsulated by ZIF-8 to form SSe@ZIF-8 (186.8 ± 23.4 nm; Fig. 1D) with core-shell structure. 3) The largest-sized selenium nanoparticles (191.3 ± 13.6 nm, Fig. S1C) were shortened to Selenium, which and ZIF-8 form Se@ZIF-8 (308.3 ± 58.3 nm; Fig. 1E) with core-satellite like structure.

The structures of SeNPs@ZIF-8 nanoparticles were identified by energy-dispersive X-ray (EDX) elemental mapping. Compared with ZIF-8, the Se element has a stronger contrast at the high-angle annular dark-field scanning transmission electron microscopy (HAADF-STEM) image. The SSSe@ZIF-8 nanoparticles presented a rhombic dodecahedral shape as ZIF-8, and the EDX elemental mapping image indicates the homogeneous distribution of SSSe in ZIF-8 (Fig. 1F). SSe@ZIF-8 exhibited typical core-shell structure which was further confirmed by EDX elemental mapping. C, O, and Zn were uniformly distributed throughout the nanostructure while Se was only located in the core, indicating that the structure of SSe@ZIF-8 was a single SSe core encapsulated by a ZIF-8 shell (Fig. 1G). The structure of Se@ZIF-8 was revealed by EDX elemental mapping that four ZIF-8 nanoparticles surround a Se core to form core-satellite like structure (Fig. 1H). For crystal structure analysis based on X-ray diffraction (XRD) pattern, SSSe@ZIF-8, SSe@ZIF-8, and Se@ZIF-8 had many overlapping peaks with pure ZIF-8, indicating that the doping of Se does not change the crystalline structure of ZIF-8. Meanwhile, their spectra had obvious peaks at 2θ angles of 23.5°, the main diffraction peaks of Se, suggesting the successful loading of selenium nanoparticles (Fig. 1I). Fourier transform infrared spectroscopy (FTIR) of selenium nanoparticles showed a characteristic vibration band. The spectra of SSSe@ZIF-8, SSe@ZIF-8, and Se@ZIF-8 was roughly consistent with that of ZIF-8, and the peak of COO^-^ at 1401 and 1602 cm^-1^, and O-H at 3400 cm^-1^ could be identified (Fig. 1J). As shown in Fig. 1K and Fig. S1D, the presence of SeLb and ZnLb peaks in the energy spectrum analysis of three types of SeNP@ZIF-8 further confirmed the successful synthesized composite nanomaterial with Se and ZIF-8. These results suggest the successful synthesis of three different construction of SeNP@ZIF-8 nanoparticles.

### Evaluation of ROS scavenging efficiency of different structural SeNPs@ZIF-8

**Fig. 2:**
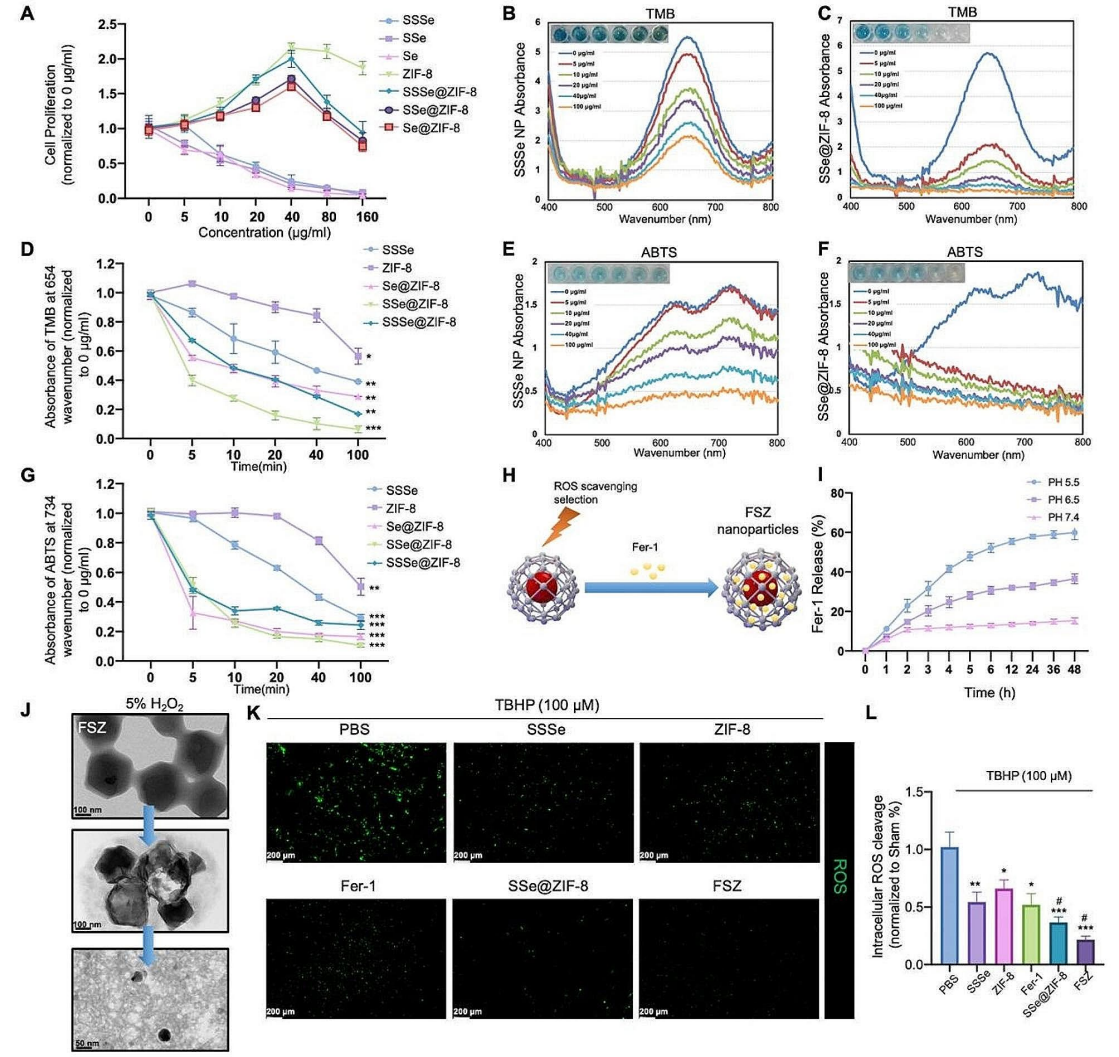
ROS scavenging effect of FSZ nanomaterials. (**A**) PC12 cells were treated with different dose (0, 5, 10, 20, 40, 80, and 160 μg/ml) of SSSe, SSe, Se, ZIF-8, SSSe@ZIF-8, SSe@ZIF-8 and Se@ZIF-8 for 48 h. CCK-8 assay was used to determine cell viabilities. (**B**-**C**) The concentration-dependent analysis (0, 5, 10, 20, 40 and 100 μg/ml) of SSSe, ZIF-8, SSSe@ZIF-8, SSe@ZIF-8 and Se@ZIF-8 was co-cultured with 100 μM H_2_O_2_, the TMB assay was performed to detect •OH elimination. (**D**) The time-dependent analysis of the absorbance of TMB at 645 nm due to •OH elimination. (**E**-**F**) The concentration-dependent investigation of ABTS•+ in the presence of SSSe, ZIF-8, SSSe@ZIF-8, SSe@ZIF-8 and Se@ZIF-8 (0, 5, 10, 20, 40 and 100 μg/ml). (**G**) The time-dependent investigation of the absorbance of ABTS•+ at 734 nm. (**H**) SSe@ZIF-8 was coated with Ferrostatin 1 to obtained FSZ nanoparticles. (**I**) Ferrostatin 1 release rate detection. (**J**) FSZ nanoparticles were co-cultured with 5%H_2_O_2_ and detected by TEM every 2 h. (**K**) PC12 cells were co-cultured using 100 μM TBHP and PBS, SSSe, ZIF-8, Fer-1, SSe@ZIF-8, and FSZ nanoparticles. Intracellular ROS level was detected by DCFH-DA probes (green) and quantitated analysis (**L**). Data are presented as means ± SD (*n* = 3). Statistical analysis was performed using one-way ANOVA. *P < 0.05, **P < 0.01, and ***P < 0.001, compared with the control group. # *P* < 0.05 compared with the SSSe group;

To evaluate the toxicity of SeNPs, ZIF-8, and SeNPs@ZIF-8, PC12 cells were treated with different concentrations of nanomaterials for 48 h. As shown in Fig. 2A, with the increase of doses, all SeNPs of different sizes showed significant cytotoxicity. However, ZIF-8 significantly promoted the proliferation of PC12 cells. Interestingly, three kinds of SeNPs@ZIF-8 promoted the proliferation of PC12 cells in the range of 0–40 ug/ml. As the concentration increased (80–160 ug/ml), the cell viabilities of PC12 cells were significantly inhibited, which may be caused by higher selenium levels in the medium.

To evaluate the antioxidant action of these nanocomposites, free radical scavenging assays in vitro were conducted. 3,3,5,5-tetramethylbenzidine (TMB) was used to detect the ability to eliminate hydroxyl radicals (•OH). Since the ROS scavenging ability of SeNPs was enhanced with decreasing size, SSSe nanoparticles were selected as the control [30]. As expected, SSSe, ZIF-8 and three different structures of SeNPs@ZIF-8 showed •OH scavenging activity (Fig. 2B-C and S2A-C). The characteristic absorption peak of TMB at 654 nm was significantly reduced in a time- and dose-dependent manner, especially SSe@ZIF-8 (Fig. 2D), which showed significant effect than other nanoparticles. 2,2′-Azinobis-(3-ethylbenzthiazoline-6-sulphonate) (ABTS) was used to test total antioxidant capacity. Similarly, SSSe@ZIF-8, SSe@ZIF-8 and Se@ZIF-8 significantly inhibited the formation of ABTS free radicals in a time-dependent manner, demonstrating a higher antioxidant capacity than SSSe and ZIF-8 (Fig. 2E-G and S2D-F). These results suggested that the capping of ZIF-8 significantly promoted the ROS scavenging ability of SeNPs, and SSe@ZIF-8 showed the better effects than other nanoparticles [23].

### Synthesis and antioxidant ability detection of FSZ NPs

ROS caused an imbalance in iron metabolism to induce neuron ferroptosis by damaging mitochondria. Fer-1 is a recognized inhibitor of ferroptosis and has been shown to maintain iron level in central nervous injury, protect the apoptosis of damaged neurons, and improve motor function impairment caused by SCI [31–33]. Therefore, the SSe@ZIF-8 nanoparticles were loaded with Fer-1 (FSZ) to achieve the inhibiting function of ferroptosis (Fig. 2H). Since the local microenvironment of SCI is near PH 6.5, the drug release rate of FSZ NPs were tested at acidic (5.5), weakly acidic (6.5) and neutral (7.4) PH values [34]. FSZ exhibited stable and controllable drug release capabilities at 6.5 and 7.4 PH values (Fig. 2I). The morphological changes of FSZ NPs after reaction with H_2_O_2_ were observed by transmission electron microscopy. As shown in Fig. 2J, FSZ NPs gradually broke down after incubation with 5% H_2_O_2_ for 2 h. In the following 4 h, FSZ was cleaved to form SeNPs monomer, indicating that FSZ NPs could degrade in 5% H_2_O_2_ environment. These results indicate that FSZ NPs have excellent recyclable antioxidant properties due to the presence of SeNPs and can react repeatedly with H_2_O_2_, while releasing Fer-1.

The ROS scavenging effect of FSZ nanoparticles in cells was further investigated. 100 μM tert-Butyl hydroperoxide (TBHP) treatment was used to induced ROS accumulation in PC12 cells, while SSSe nanoparticles were set as a positive control for more ROS scavenging ability and lower toxicity than other SeNPs [35]. Intracellular ROS levels were evaluated by DCFH-DA probe. Compared with SSSe, SSe@ZIF-8 and FSZ NPs significantly reduced ROS production in TBHP-treated PC12 cells, indicating their more excellent ROS scavenging activity (Fig. 2K and L). The effect of FSZ NPs on the cell cycle was examined as SSe@ZIF-8 showed an effect of promoting cell proliferation at low doses. As shown in Fig. S3, FSZ increased the proportion of G2/M-phase cells in PC12 cells, indicating that it promoted the proliferation potential of cells at low doses (0–20 μg/ml). Above all, FSZ NPs showed excellent ROS elimination ability and good biocompatibility in vitro.

### FSZ NPs prevent apoptosis and maintain mitochondrial homeostasis

**Fig. 3:**
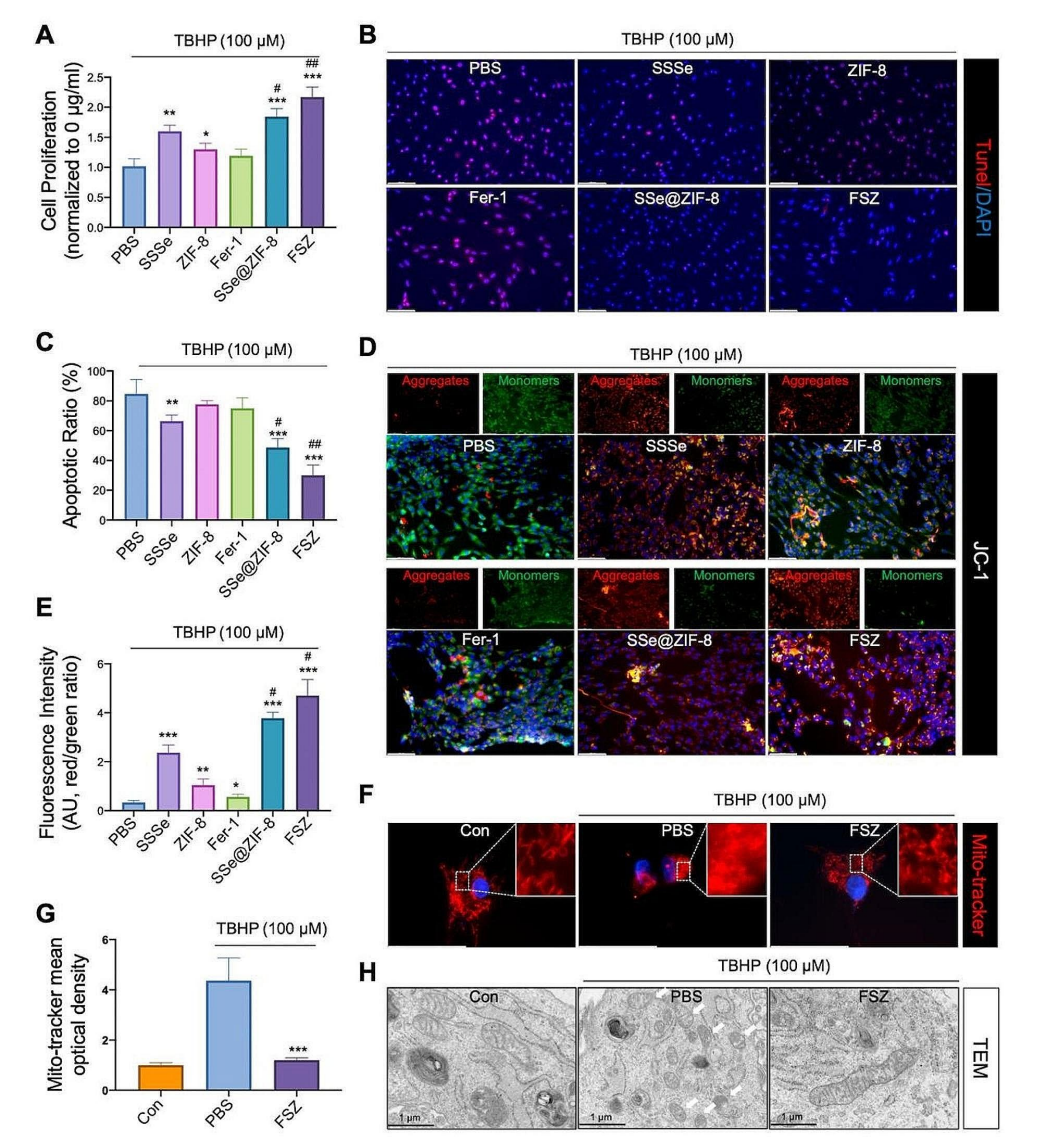
FSZ nanoparticles inhibited apoptosis and maintained mitochondrial homeostasis. PC12 cells were co-cultured using 100 μM TBHP and PBS, SSSe, ZIF-8, Fer-1, SSe@ZIF-8, and FSZ nanoparticles. (**A**) CCK-8 assay was used to determine cell viabilities. (**B**) TUNEL staining was used to detect apoptosis (red). (**C**) Tunel-positive cells were quantified. (**D**) Measurement of mitochondrial membrane potential was performed with JC-1 staining. The JC-1 monomer (Green) accumulates in mitochondria, indicating that the membrane potential decreased. (**E**) Quantification of mitochondrial membrane potential after staining (Red/Green). (**F**) PC12 cells were stained with Mito-tracker Red, which is a mitochondrial superoxide indicator, and (**G**) the fluorescence intensity of MitoSOX was quantified. (**H**) Ultrastructure of the PC12 cells were co-treated with or without FSZ nanoparticles and 100 μM TBHP. Statistical analysis was performed using one-way ANOVA. Scale bar: 100 μm. *P < 0.05, **P < 0.01, and ***P < 0.001, compared with the PBS group; #*P* < 0.05, and ##*P* < 0.01 compared with the SSSe group

To further explore whether FSZ NPs can prevent nerve cell apoptosis, exceed ROS was generated in PC12 by TBHP treatment. As shown in Fig. 3A, SSSe, ZIF-8, Fer-1, SSe@ZIF-8 and FSZ NPs were all able to rescue the inhibition of cell viabilities induced by TBHP. Compared with SSSe nanoparticles, SSe@ZIF-8 and FSZ have better antagonistic effects of TBHP. Given the important role of ROS in nerve apoptosis, the neuron-protective effect of FSZ NP-mediated apoptosis reduction was investigated. TUNEL staining was used to detect the total apoptotic levels in PC12 cells treated with TBHP (Fig. 3B and C). As expected, FSZ and SSe@ZIF-8 significantly inhibited the apoptosis level of PC12 cells, and their effects were significantly better than SSSe. Then, cell apoptotic distribution was performed by flow cytometry (FACS). Similar with results of cell viability and TUNEL staining, FSZ NPs showed more apoptotic inhibition induced by TBHP than SSSe or other nanomaterials (Fig. S4A and S4B). Cell cycle analysis indicated that FSZ NPs inhibited the portion of subG1 cell sets from 21.03 to 1.9% with increased concentration (0–40 μg/ml, Fig. S5). These results suggested that FSZ and SSe@ZIF-8 NPs significantly inhibited the apoptosis induced by exceed ROS.

Excessive accumulation of oxidative stress can damage the structure of the mitochondria inside the cell. ROS is produced as a by-product during aerobic respiration by mitochondria, which is closely related to the apoptosis and ferroptosis of neuron [36]. Here, we used biological TEM to detect the distribution of FSZ NPs in the cell. Although SeNPs are not exclusively present in mitochondria after degradation of FSZ, they are still enriched by mitochondria (Fig. S6). The reason maybe that mitochondrial membrane depolarization reduces its negative potential, thereby reducing the repulsion between SeNPs and mitochondria, thereby increasing the intrinsic targeting of mitochondria.

The protective effect of FSZ NPs on mitochondria of neural cells was further determined. By interfering with the mitochondrial respiratory chain, the SCI cascade increases H_2_O_2_ and ·O_2_^−^ concentrations in mitochondria. The high level of mitochondrial ROS causes depolarization of mitochondrial membrane and mitochondrial dysfunction. As shown in Fig. 3D and E, FSZ and SSe@ZIF-8 NPs significantly decreased the JC1 monomer levels, while increased the aggregates, which suggested that the nano therapy restored normal mitochondrial membrane potential. Mitochondrial morphology was tracked by Mito-tracker Red to determine the protective effect of FSZ NPs on mitochondria. As shown in Fig. 3F, the cytoplasmic red fluorescence intensity increased and mitochondria became fragmented with the treatment of TBHP, and FSZ NPs rescued the mitochondrial morphology, which suggested the decreased mitochondrial ROS levels of PC12 cells. FSZ NPs significantly scavenged excess mitochondrial ROS, which indicated the injured mitochondria was rescued (Fig. 3G). Mitochondrial morphology was also further observed in PC12 cells treated with TBHP. Compared with that in the control groups, mitochondria in PBS groups had swollen, ruptured, and lost the cristae in the inner mitochondrial membrane, which suggested that the mitochondrial homeostasis has been destroyed (Fig. 3H). In FSZ groups, the nano-therapy maintained the mitochondrial shapes. Together, FSZ NPs with a non-toxic dose protects neuronal mitochondrial and prevents cells from apoptosis and ferroptosis induced by exceed ROS due to its free radical scavenging efficiency.

### FSZ NPs inhibit neural inflammation

SeNPs has the ability to inhibit the expression and release of inflammatory factors by regulating mitochondrial homeostasis. Therefore, we examined the ability of FSZ to promote cell polarization in macrophages. LPS (1 μg/mL) was used to induce RAW264.7 cells in the inflammatory state that the expression of iNOS was increased, while the expression of Arg1 was decreased (Fig. S7A). Western blotting showed that the changes in expression of these proteins were reversed by SSe@ZIF-8 and FSZ NPs (Fig. S7B), which indicated that the ratio of M2 cells was promoted. To further detect changes in the expression of inflammatory factors, TNF-α and IL-10 was investigated. SSe@ZIF-8 and FSZ NPs significantly inhibited the TNF-α expression, and increased IL-10 levels, suggesting the inflammatory inhibitory effects (Fig. S7C). To further identify the phenotypic change of RAW264.7 cells under the treatment of FSZ NPs, Flow CytoMetry (FCM) was used to show the proportion of M1 (CD86 + and CD206-) and M2 (CD86- and CD206+) macrophages. As shown in Fig. S8A and S8B, FSZ NPs exhibited inhibition of M1 polarization and promotion of M2 polarization. The similar results were further identified with IFS (Fig. S9A and S9B). These results suggest that FSZ NPs can regulate the transformation of macrophages from M1 phenotype to M2 phenotype, providing a low-inflammation microenvironment for neural repair through protecting mitochondrial homeostasis.

### FSZ NPs promotes neurite outgrowth and neuronal maturation

**Fig. 4:**
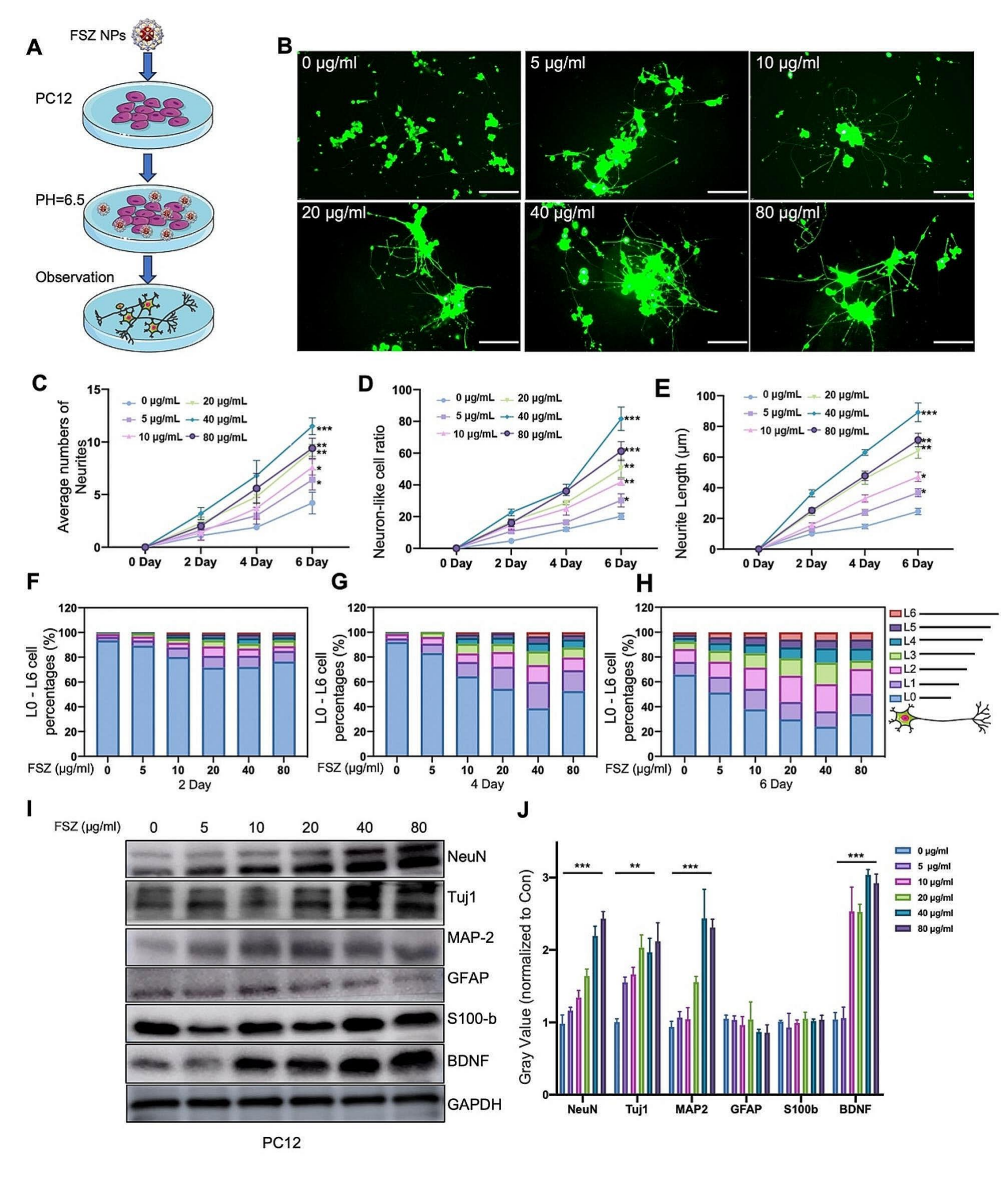
Property of axonal growth promotion by FSZ nanoparticles. (**A**) Schematic illustration of the co-culture of PC12 cells and FSZ nanoparticles. (**B**) Fluorescence images of Calcein AM-labeled PC12 cells co-cultured with with FSZ (0, 5, 10, 20, 40 and 80 μg/ml) for 6 d. Scale bar: 100 μm. (**C**-**E**) The neurite number, neurite ratio, and neurite length of PC12 cells co-cultured with FSZ (0, 5, 10, 20, 40 and 80 μg/ml) for 2, 4, and 6 days. (**F**-**H**) The growth radio of PC12 cells incubated with FSZ (0, 5, 10, 20, 40 and 80 μg/ml) for 2, 4, and 6 days. The level of growth was quantitatively divided into L0 to L6 according to the length of the neurites. Ln means that the longest neurite length is n to *n* + 1 times the diameter of the cell body. (**I**) Protein levels of NeuN, Tuj1, MAP-2, GFAP, S100-b, BDNF and GAPDH in PC12 cells incubated with FSZ (0, 5, 10, 20, 40 and 80 μg/ml) for 6 days determined by Western blot. Protein levels were quantified using ImageJ (1.53a for Mac). Data are presented as means ± SD (*n* = 3). Statistical analysis was performed using one-way ANOVA. *P < 0.05, **P < 0.01, ***P < 0.001

Our previous work indicated that ZIF-8 promoted the neurodifferentiation ability of MSCs by releasing zinc ion [26]. In order to detect the effects of this composite nanomaterial on neural differentiation function, a co-culture system was designed to compare the effects of different concentrations (0, 5, 10, 20, 40, and 80 μg/ml) of FSZ on the promotion of PC12 neural differentiation. Due to the acidic microenvironment of the SCI, after FSZ entered the cells, the medium was replaced with a slightly acidic environmental (PH = 6.5) with neuro-induction condition (Fig. 4A), which facilitated the breakdown of ZIF-8 NPs to release Zn^2+^. The neurite length of the newborn neurites and each-stage differentiated cell was monitored every 2 days. Figure 4B shows a representation image executed at the end point for all groups. With the increased concentration of FSZ, the average number of new neurites per differentiated cell increased significantly at the range of 0–40 μg/ml (Fig. 4C). Similarly, the differentiation rates were upregulated from with 20–80% with the increased concentration of FSZ (0–40 μg/ml) (Fig. 4D). Neurite length also increased significantly, especially at concentrations of 40 μg/ml, and their average length was 4 times than that of the control group (Fig. 4E). We divided differentiated PC12 cells into seven groups (from L0 to L6) based on the ratio of neurite length to the corresponding cell body length from previous work [37]. Neurite elongation increased slowly within 2 days after treatment of FSZ NPs, while there was no significant increase in the control group (Fig. 4F). After day 4, this increase accelerated abruptly (Fig. 4G). At the end of day 6, the L0% in the control group was 60%, while the L0% in the 40 μg/ml SSe@ZIF-8 decreased to nearly 30%, and the percentage of highly differentiated cells (L3 to L6) increased with the increase of FSZ NP concentration (Fig. 4H). As shown in Fig. 4I and J, the expression of NeuN (neuron marker), βIII-tubulin (mature axon markers), MAP-2 (mature axon markers) and brain-derived neurotrophic factor (BDNF) was significantly up-regulated with the increase of FSZ concentration, as well as the down-regulated expression of GFAP (astrocyte marker) or no impact of S100b (astrocyte and Schwann cell markers). Above all, FSZ NPs directly promoted neuronal differentiation and inhibited glial cell formation.

An interesting phenomenon is that FSZ particles can promote the differentiation of undifferentiated PC12 cells even in normal medium (non-neural induction). As shown in Fig. S10A, normal undifferentiated PC12 cells should be in a suspended clump state. After 6 days of treatment, these undifferentiated PC12 cells showed a number of low differentiated state with significant morphologic change and axon extension, while with the manifestation of cell dispersion adhesion. The proportion of poorly differentiated PC12 cells gradually increased with the increase of FSZ concentration, reaching the highest level of 22% at 40 μg/ml (Fig. S10B), which suggested FSZ NPs may promotes neuronal maturation. However, the lack of further induction conditions prevents the progression of this differentiation phenomenon [38].

The local delivery of the nanomaterial and drug strategy has fewer side effects and higher concentration than intravenous administration [39]. Therefore, we plan to apply FSZ NPs in the local treatment of acute SCI with surgery to simulate the real clinical application scenario. However, a problem to be faced is that as SCI destroys the blood-spinal barrier, large amounts of cerebrospinal fluid and blood escape may lead to dilution and escape of nanomaterials, reducing therapeutic effectiveness [40]. Therefore, we selected GelMA hydrogel, a biological scaffold material with high biocompatibility, to immobilize FSZ NPs locally in SCI injury to overcome the above problems [41]. To evaluate the cell recruitment effect of FSZ-GelMA, we constructed an in vitro model of local application (Fig. S11A). PC12 cells were cultured in the upper compartment, while medium in the lower compartment was serum-free to expel the attraction and proliferation effect of serum on PC12 cells. After 3 days of culture, the GelMA hydrogel was degraded and the number of PC12 cells in the medium of the lower chamber was quantified. As shown in Fig. S11B, with the increase of FSZ concentration, the number of PC12 cells crossing the barrier increased, reaching three times that of the control group at 20 μg/ml.

### FSZ promotes neural differentiation by activating the WNT4/β-catenin pathway

**Fig. 5:**
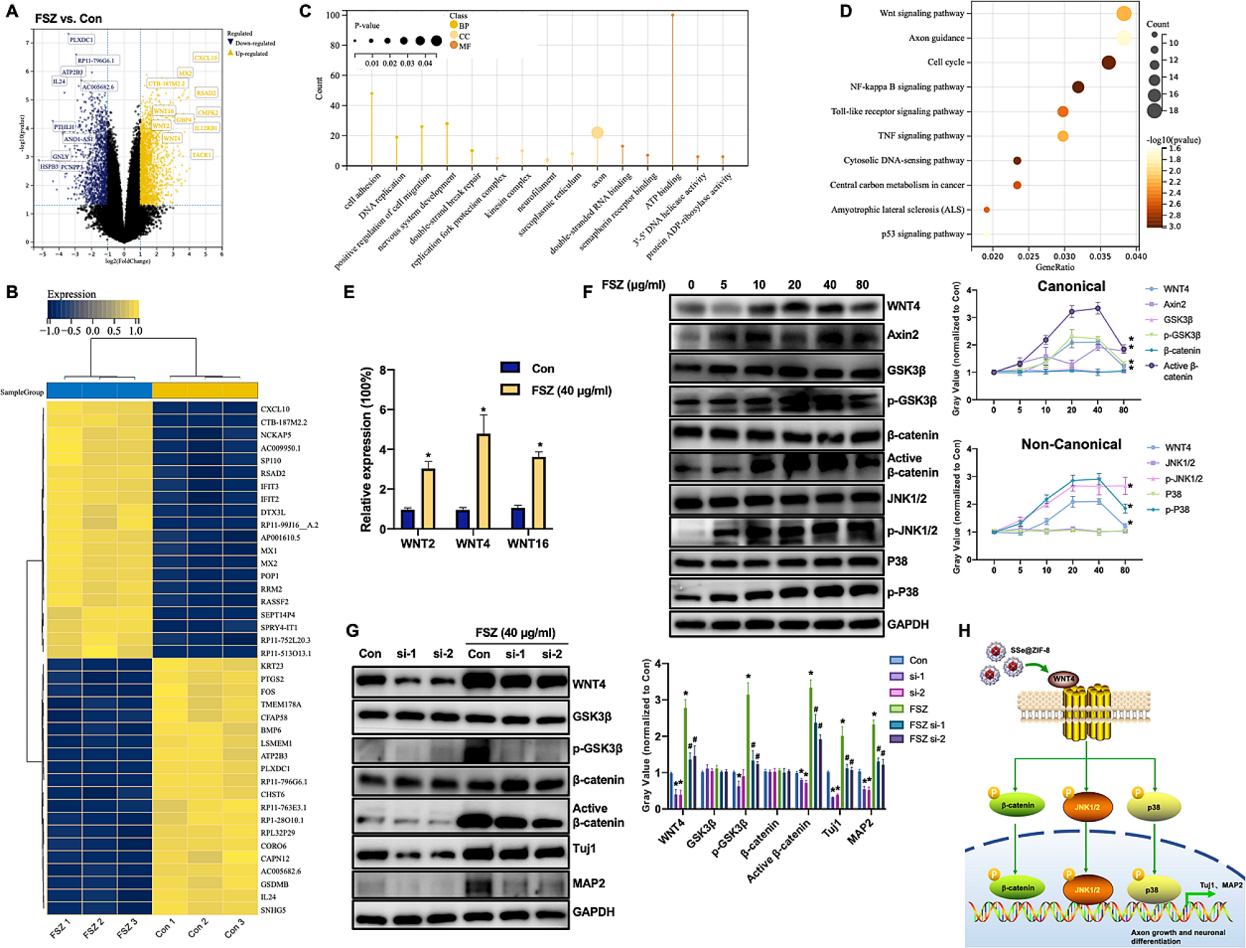
FSZ NPs promote neural differentiation through the WNT4 signaling pathway. MSCs were treated with FSZ (0 and 40 μg/ml) for 6 days in neural inducing medium, total mRNA was extracted and subjected to RNA-sequence assay for gene expression identifying. (**A**) Volcano maps and (**B**) heat maps are displayed. (**C**) GO and (**D**) KEGG analyzed the up-regulated genes associated with FSZ. (**E**) MSCs were treated with FSZ (0, 5, 10, 20, 40 and 80 μg/ml) for 6 days in neural inducing medium, Western blot assay was used to detect the expression of WNT4, Axin2, GSK3β, p-GSK3β, β-catenin, active-β-catenin, JNK1/2, p-JNK1/2, P38, p-P38 and GAPDH. (**F**) MSCs were treated with FSZ (0 and 40 μg/ml) combination with transfection of WNT4-siRNA1 or WNT4-siRNA2 for 6 days in neural inducing medium, Western blot assay was used to detect the expression of WNT4, GSK3β, p-GSK3β, β-catenin, active-β-catenin, Tuj1, MAP2 and GAPDH. Protein levels were quantified using ImageJ (1.53a for Mac). Data are presented as means ± SD (*n* = 3). Statistical analysis was performed using one-way ANOVA. # *P* < 0.05, compared with FSZ group; * *P* < 0.05 compared with Con group

To explore how FSZ NPs promote the effect of neural differentiation, RNA-seq technology was used to widely screen for changes in gene expression affected by FSZ. As shown in Fig. 5A, a total of 2054 genes were up-regulated, accompanied by 1699 genes down-regulated (Log2FC > 2, *P* < 0.05). Heat maps show significant clusters of the top 40 gene groups (Fig. 5B). These upregulated genes are mainly concentrated in cell adhesion, DNA replication, nervous system development, neurofilament, axon, positive regulation of cell migration and other cell functions (Fig. 5C). KEGG enrichment analysis showed that these genes were mainly related to WNT signaling pathway, cell cycle, NK-κB signaling pathway, etc. (Fig. 5D). Several members of the WNT family were found in upregulated gene clusters, including WNT2, WNT4, and WNT16 (Fig. 5E).

As shown in Fig. 5F, with the increase of FSZ nanomaterial concentration, the expressions of WNT4, Axin2, p-GSK3β and active-β-catenin were significantly up-regulated, while the expressions of GSK3β and β-catenin remained unchanged. This suggests that FSZ activates both typical and WNT4 signaling pathways in MSCs. In addition, the expression of p-JNK1/2 and p-p38 was also significantly increased, suggesting that the non-classical WNT4 signaling pathway was also activated. In order to explore whether the activation of WNT4 signaling pathway is related to axon growth, small interfering RNA of WNT4 combined with FSZ was co-cultured with MSCs cells. As shown in Fig. 5G, si-WNT4 (si-1 and si-2) significantly inhibited WNT4 expression, while p-GSK3β and active-β-catenin expression were also inhibited. As expected, Tuj1 and MAP2 expression decreased with si-WNT4 transfection. These results suggest that FSZ NPs promote cellular neural differentiation by activating WNT4-dependent (canonical and non-canonical) signaling pathways (Fig. 5H).

### FSZ promotes angiogenesis by activating VEGF expression

ZIF-8 enhances its angiogenesis ability by promoting VEGFa expression in MSCs [42]. The influence of FSZ NPs on blood vessel formation was explored with human umbilical vein endothelial cells (HUVECs) and PC12 cells. We first examined whether VEGFa expression in PC12 cells was affected by FNZ NPs treatment. As shown in Fig. S12A and S12B, FSZ significantly promoted VEGFa expression. Meanwhile, ELISA assay indicated that FSZ NPs increased the concentration of VEGF-a in supernatant suggesting the secretion of VEGF-a was enhanced (Fig. S12C). Subsequently, the supernatant of PC12 cells was used to co-culture with HUVEC to accomplish the tube formation experiment. HUVECs were stained with Calcein AM and their morphology was observed (Fig. S13A). It was found that FSZ NPs significantly promoted the formation of capillary-like structures in a dose-dependent manner. The number of notes, meshes and capillary length were significantly increased in the 40 μg/mL FSZ NPs group compared with the control group, suggesting strong pro-angiogenic activity (Fig. S13B-S13D). In addition, a trans-well experiment suggested that the conditional medium of PC12 cells with FSZ NPs significantly promoted the number of HUVECs across the barrier by a dose-dependent manner (Fig. S14A and S14B), which suggested the enhanced vascular endothelial cell migration ability from damaged BSCB. These results suggest FSZ NPs has the bioactivity of promoting angiogenesis, which is benefit to the post-injury microenvironment to regulating inflammation and ROS scavenging.

### Dual invasion mechanisms of FSZ NPs

**Fig. 6:**
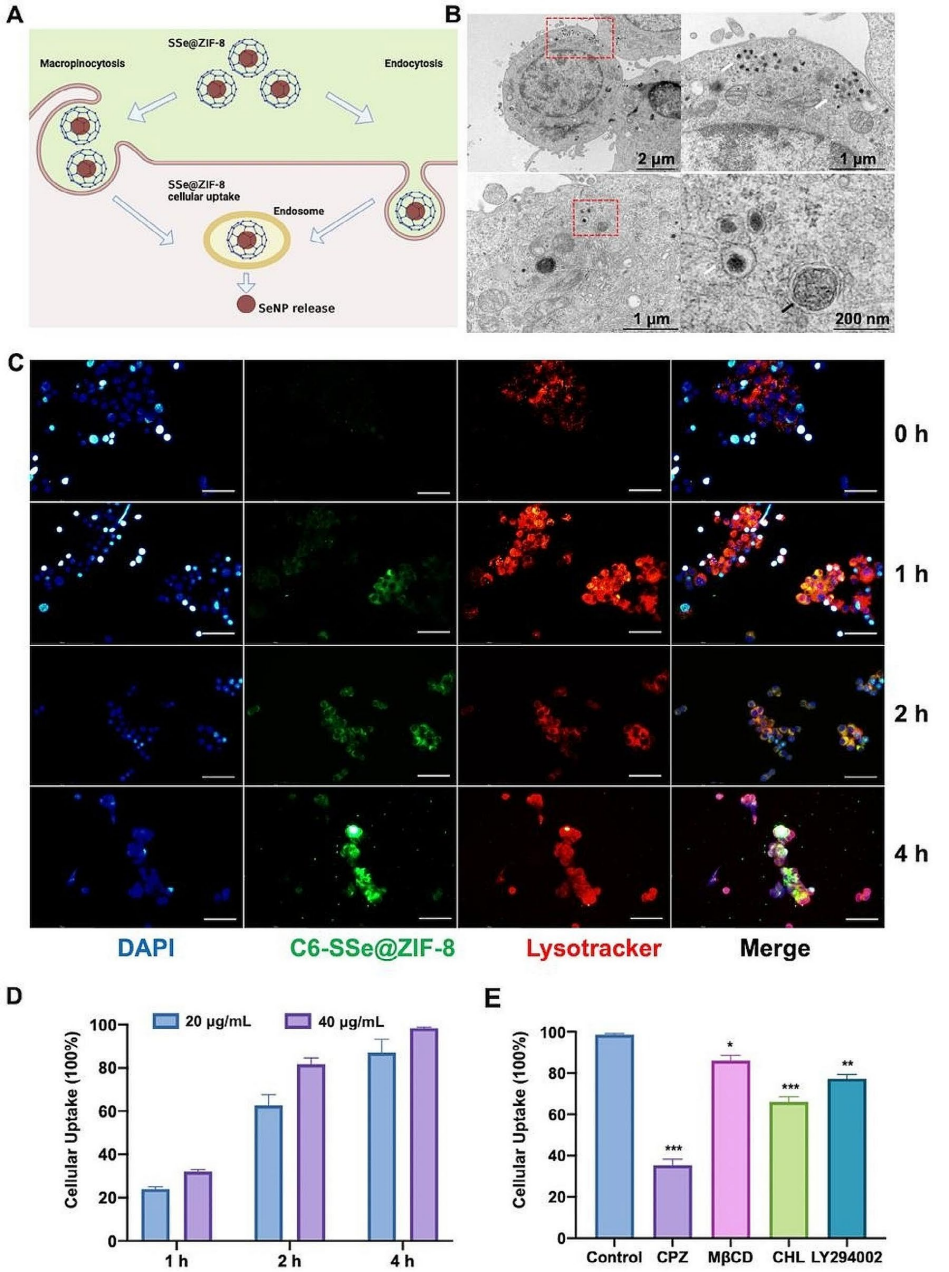
Internalization mechanism of SSe@ZIF-8. (**A**) Schematic illustration of internalization of SSe@ZIF-8. (**B**) TEM images of PC12 cells treated with SSe@ZIF-8 for 6 h. white arrow: SSe@ZIF-8 in lysosomes; black arrow: SSe@ZIF-8 in autophagic vesicles. (**C**) CLSM images of intracelluar uptake of SSe@ZIF-8 at 0, 1, 2, and 6 h. SSe@ZIF-8 (C6: green), lysosomes (Lyso Tracker: red), and nuclei (DAPI, blue). Scale bar: 100 μm. (**D**) Quantitative analysis of intracellular uptake of SSe@ZIF-8 in PC12 cells. (**E**) Intracellular uptake of SSe@ZIF-8 in PC12 cells inhibited by different endocytosis inhibitors. Chlorpromazine (CPZ, an inhibitor of CDE), Methyl-β-cyclodextrin (MβCD, an inhibitor of CIE), chloroquine (CHL, an inhibitor of CDE), and LY294002 (an inhibitor of macropinocytosis). Data are presented as means ± SD (*n* = 3). Statistical analysis was performed using one-way ANOVA. *P < 0.05, **P < 0.01, ***P < 0.001

The ability of nanoparticles to trigger biological responses is closely related to the way they internalize in cells [43]. Pinocytosis is an important mechanism for nanomaterials to enter cells, which is commonly classified into phagocytosis, endocytosis (clathrin-dependent, caveolae-dependent, or clathrin/caveolae-independent endocytosis) and macropinocytosis [44, 45]. To investigate cellular uptake of SSe@ZIF-8 NPs complexes (Fig. 6A), we tracked the intracellular distribution and pathway of the nanocomplexes by transmission electron microscopy (TEM) after PC12 cells treated with nanoparticles for 6 h. As shown in Fig. 6B, SSe@ZIF-8 NPs were coated with vesicles. Interestingly, images of typical endocytosis (Fig. S15A) and macropinocytosis cups (Fig. S15B) are captured as the nanoparticles enter the cell. Both in endocytosis and macropinocytosis pathways, allogeneic materials are delivered from the plasma membrane to the early endosomes [46, 47]. Subsequently, some of them are recycled to the cell surface as circulating endosomes, while others gradually mature into late endosomes and are eventually transported to lysosomes, so there is a massive accumulation of cargo in the lysosomes [48]. SeNPs were found in the lysosome of the cell instead of FSZ nano-polyhedras (Fig. 6B). To further confirm the cellular uptake process and mechanism of SSe@ZIF-8 NPs, SSe@ZIF-8 was labeled with Coumarin 6 (C6) and the lysosomes were stained with the fluorescent tracker, LysoTracker (Fig. 6C). Immunofluorescence showed that the green fluorescence of C6-labeled SSe@ZIF-8 nanoparticles were gradually entering the cells in 1 h, and well co-localized with the red fluorescence of lysosomes in 2 h. At 4 h, these nanopolyhedra and lysosomes underwent strong colocalization. Figure 6D showed the efficiency of cellular uptakes.

To further confirm the internalization pathway of SSe@ZIF-8, we used different endocytosis inhibitors, including Chlorpromazine (CPZ, an inhibitor of CDE), Methyl-β-cyclodextrin (MβCD, an inhibitor of CIE), chloroquine (CHL, an inhibitor of CDE), and LY294002 (an inhibitor of macropinocytosis). The cellular uptake efficiency of SSe@ZIF-8 was significantly reduced by CPZ, MβCD, CHL and LY294002. (Fig. 6E). Especially for CPZ, the internalization efficiency was inhibited with a reduction at 70%, which suggested that clathrin-dependent endocytosis played the major role in internalized process of SSe@ZIF-8 NPs. Above all, both lysosome-mediated macropinocytosis and endocytosis pathways participate internalization of SSe@ZIF-8 nanoparticles.

### Motor-functional rescuing of SCI rats by local administration of FSZ NPs

**Fig. 7:**
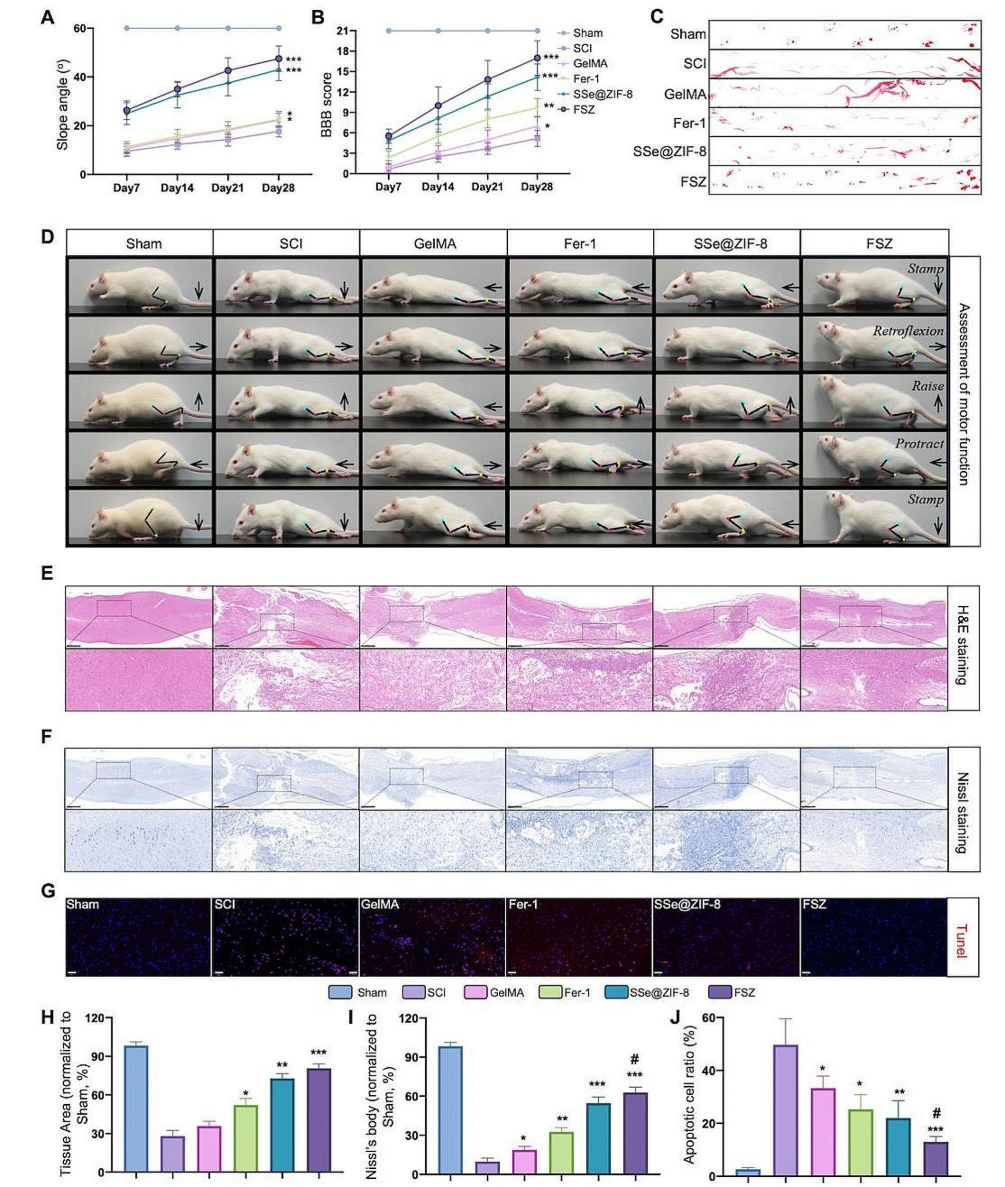
FSZ NPs promote the recovery of motor function in SCI rats. (**A**) Slope test of mice in each group at the corresponding time points. Largest Slope angles that mice can stay for 5 min were recorded. (**B**) BBB of mice in each group at the corresponding time points. (**C**) The gait marks of the mice in each group at Day 28. (**D**) Video analysis showing the motion of the mice in each group. The green, red, and yellow dots represent the hip, knee, and ankle joints, respectively. (**E**) H&E staining images of spinal cord in the injury site of mice in each group. Scale bar: 500 μm. (**F**) (**G**) Tunel staining (red) images of spinal cord sections. Scale bar: 100 μm. Nissl staining images of spinal cord in the injury site of mice in each group. (**H**) Quantitative analysis of the H&E staining images of spinal cord in the injury site. (**I**) Quantitative analysis of the Nissl staining images of spinal cord in the injury site. (**J**) Quantitative analysis of apoptotic cell stained by Tunel (red). Data are presented as means ± SD (*n* = 3). Statistical analysis was performed using t-test. * *P* < 0.05, ** *P* < 0.01, and *** *P* < 0.001 compared with SCI group; # *P* < 0.05, compared with SSe@ZIF-8 group

In order to clarify the role of FSZ NPs in protecting tissue damage caused by acute SCI in vivo, we constructed a rat model of acute SCI. The combination strategy of FSZ loaded with GelMA hydrogel was used to treat SCI. The motor function of the hind limbs of rats was evaluated by video recordings, Basso, Beattie & Bresnahan (BBB) Locomotor Rating Scale, oblique plate test, and footprint analysis on Day 7, 14, 21 and 28 post-injuries. In the slope experiment, there was no significant difference between groups in the angles at which rats could be stay for 5 s in the early stage. On Day 28, The hind leg bearing capacity of the SSe@ZIF-8 and FSZ NPs group was significantly stronger than that of the other groups (Fig. 7A). The BBB score of FSZ NPs group was significantly improved compared with the control group, followed by that of the SSe@ZIF-8 and Fer-1 group (Fig. 7B). In footprint analysis (Fig. 7C), the foot pads of the SCI and GelMA groups showed obvious dragging marks, those of the Fer-1 group showed occasional supporting marks, and those of the SSe@ZIF-8 group was observed some footprints. FSZ NPs exhibited two orderly rows of footprints, albeit with much shorter steps than the Sham group. As shown in the video, on Day 28 after surgery, the rats in the SCI group had no obvious hind limb movement, the rats in the GelMA and Fer-1 groups could reclaim their hind limbs, and the rats in the SSe@ZIF-8 and FSZ groups could step forward (Fig. 7D). Compared with the SCI group and other treatment groups, rats in FSZ NPs group had higher height from the ground (Fig. S16A), less foot error (Fig. S16B), shorter spasm duration (Fig. S16C) and longer forward distance (Fig. S16D). Together, these results suggest that FSZ and SSe@ZIF-8 nanoparticles significantly promote the recovery of motor function in SCI rats.

### FSZ NPs promotes neural regeneration and prevents glial scar formation in vivo

Although local application of nanomaterials leads to fewer side effects, we tested major organs throughout the body of rats to determine their biocompatibility. As shown in Fig. S17A, the sections of major organs of rats, including heart, liver, spleen, lung, and kidney, were stained with H&E. The results presented no obvious inflammation infiltration or any other pathological injury after treatment in FSZ and other groups. In addition, the blood routine and biochemical tests also indicated that the liver and kidney function of the rats were normal, and there were no other inflammatory reactions (Fig. S17B). Together, these results suggest that local application of FSZ at this concentration provides good biocompatibility.

**Fig. 8:**
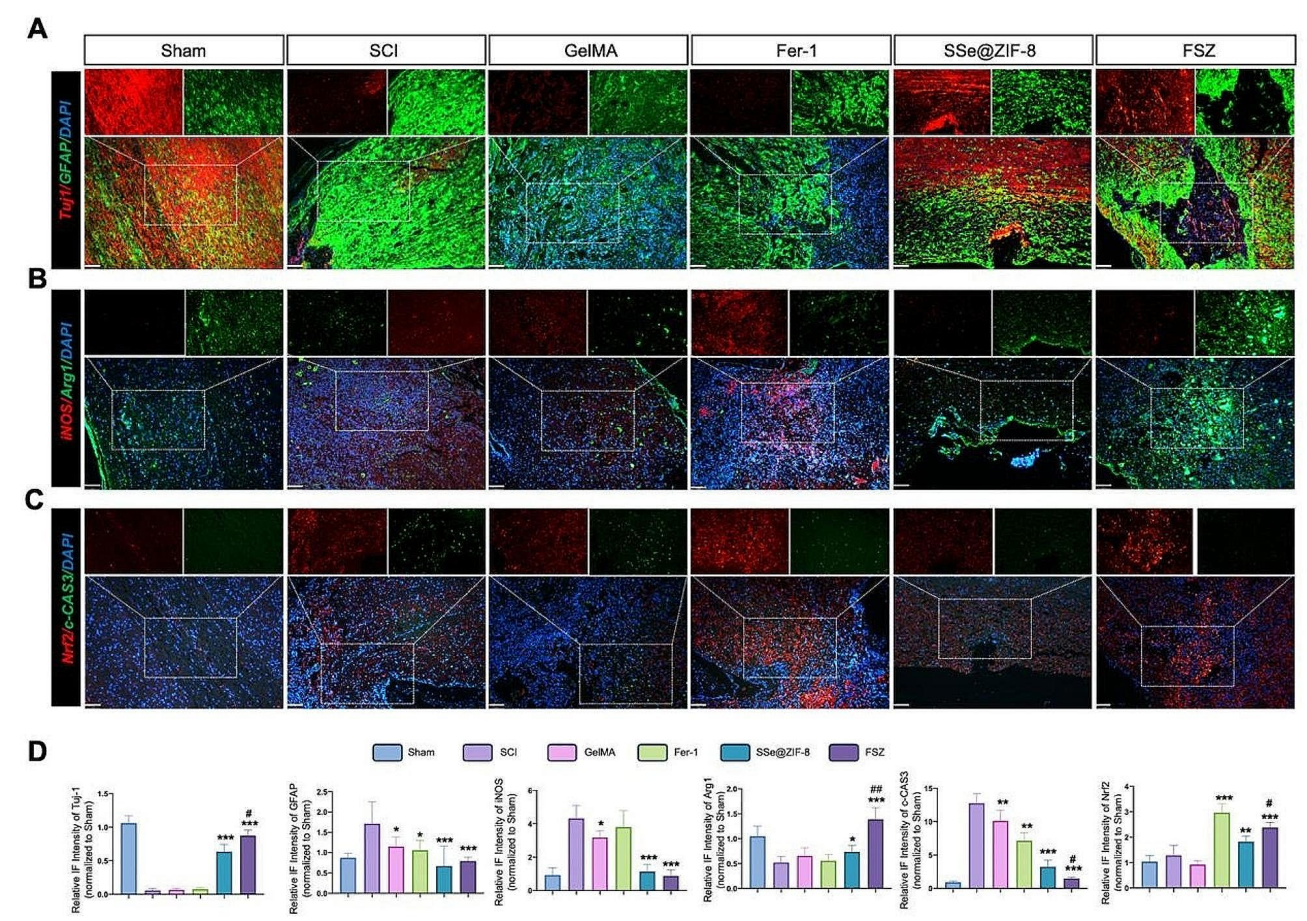
FSZ NPs exhibit versatile biological properties for neural regeneration in lesion sites. (**A**) Immunofluorescence images of spinal cord sections. Neurons (Tuj-1, red), Glial cells (GFAP, green) and DAPI-nuclei (blue). Scale bar: 100 μm. (**B**) Immunofluorescence images of spinal cord sections in each group. M1 (iNOS: red), M2 (Arg1: green), and nuclei (DAPI: blue). Scale bar: 100 μm. (**C**) Immunofluorescence images of spinal cord sections in each group. NRF2 (red), c-CAS3 (green) and DAPI (blue). Scale bar: 100 μm. **D**-**E**) Quantitative analysis of the expression of (**D**) Quantitative analysis of the expression of Tuj-1, GFAP, iNOS, Arg1, NRF2, and c-CAS3. Scale bar: 100 μm. Statistical analysis was performed using one-way ANOVA. *P < 0.05, **P < 0.01, ***P < 0.001; # *P* < 0.05, compared with SSe@ZIF-8 group

Next, we explored the repair effect of FSZ NPs on spinal cord tissue after injury. H&E staining of injured nerves demonstrated that the spinal cord in the FSZ NPs group had less cavities and scar and better density and continuity (Fig. 7E and H). Interestingly, pathological results showed that small tissue cavities were still preserved in SSe@ZIF-8 and FSZ groups. Nissl staining showed that the spinal cord in the FSZ NPs group had more Nissl’s bodies, indicating the neurons were metabolically active (Fig. 7F and I). TUNEL staining showed that apoptosis in the Fer-1, SSe@ZIF-8 and FSZ groups were reversed compared with the SCI group (Fig. S7G and 7 J). However, FSZ NPs has a better anti-apoptotic effect than SSe@ZIF-8 NPs, which was consistent with the results in vitro. A double staining with Tuj-1 (βIII-tubulin, marker of neurons) and GFAP (marker of glial cells) were performed to detect neural regeneration and glial scar in lesion sites. As shown in Fig. 8A, significant glial scars and less axons could be found in injury center of SCI, GelMA and Fer-1 groups. Interestingly, astrocytes uniformly surrounded the injured center in SSe@ZIF-8 and FSZ groups, suggesting the glial scar formation was significantly prevented (Fig. 8D). In addition, a large number of axons were induced gathering toward or cross the injury center (Fig. 8A and D), which indicated potential regeneration of neural circuits. This explains that in the SCI, gelMA, and Fer-1 groups, the tissue recovery center was still dominated by glial scars, while SSe@ZIF-8 and FSZ NPs preserved the space for neuronal regeneration. SCI is associated with a large loss of Zn ions, which indirectly prevents axon growth and NSC differentiation [49]. By TSQ staining, we can observe that the level of Zn^2+^ in the injured sites of SCI, GelMA and Fer-1 groups was lower. The treatment of SSe@ZIF-8 and FSZ restored Zn ion levels in the nerve cells (Fig. S18A and S18B). Interestingly, some strong Zn ion aggregation signals were detected, suggesting that some undecomposed nanoparticles remain in the damaged tissue, providing evidence for sustained SCI treatment (Fig. S18A, white arrow). By releasing Zn^2+^, SSe@ZIF-8 and FSZ NPs could promote the growth of neurons and axons toward the injury center. Importantly, these results suggest that SSe@ZIF-8 and FSZ NPs can exist in SCI injured tissue in response to prolonged neural regeneration and recovery.

### FSZ NPs regulates macrophage polarization and promotes angiogenesis

By clearing mitochondrial ROS accumulation and regulating inflammatory factor expression, FSZ effectively promoted macrophage differentiation to M2 phenotype in vitro. Neuroinflammation in spinal cord injury was also evaluated. Compared with SCI group, Arg1 was expressed aggregately in lesion center of SCI in FSZ groups, with reduced level of iNOS, which suggested that FSZ contribute the transformation of M0 macrophages to M2 macrophages (Fig. 8B and D). Through the balance of mitochondrial homeostasis, macrophage polarization and the expression of inflammatory factors affect each other. FSZ and SSe@ZIF-8 inhibited TNF-α and IL-6 levels, while increased IL-4 expression in serum of SCI rats (Fig. S19A-S19C), which indicated the anti-inflammatory effect. This combined strategy of FSZ NPs inhibited neuroinflammation and created a suitable microenvironment for spinal cord regeneration.

Excessive ROS accumulation leads to mitochondrial respiratory disorder, promotes the release of cytochrome C, activates caspase3 and leads to cell apoptosis. On the other hand, ROS also caused iron metabolism abnormalities and induced liposomal peroxidation leading to ferroptosis [50]. Compared with SCI group, IFS results suggested that FSZ NPs increased the expression of Nrf2 and decreased the expression of cleaved-Caspase3 (c-CAS3) which suggested the inhibition of ferroptosis and apoptosis, respectively (Fig. 8C and D). The angiogenetic effect of FSZ NPs is revealed by CD31 staining. In FSZ and SSe@ZIF-8 groups, the number of CD31 + cells increased significantly (Fig. S20A and S20B). These results suggest that FSZ NPs regulates local neuroinflammation and promotes angiogenesis in SCI rats.

## Discussion

In this study, a series of composite materials based on SeNPs and zeolite ZIF-8 were developed to enhance antioxidant activity and promote neural precursor cell differentiation. By controlling the size of the SeNPs, we synthesized three different structures SeNPs@ZIF-8 and confirmed their structural characteristics. These composites showed significant ROS clearance and neurodifferentiation at non-toxic doses. The medium-size SeNPs@ZIF-8 was found to be the best at clearing ROS. By loading Fer-1, an iron death inhibitor, into SSe@ZIF-8, FSZ nanoparticles showed stable drug release ability under acidic environment, and could be decompressed under oxidative stress to release SeNPs and Fer-1 with antioxidant properties. This indicated that FSZ NPs have cyclic antioxidant properties. In cell experiments, FSZ NPs effectively reduced ROS accumulation in PC12 cells, inhibited cell apoptosis, and maintained mitochondrial homeostasis. Importantly, FSZ NPs could promote the neural maturation and axonal extension of PC12 cells by activating WNT4-dependent signaling pathway. In addition, FSZ NPs could also regulate the polarization of macrophages from the pro-inflammatory M1 type to the anti-inflammatory M2 type, thus providing a favorable microenvironment for nerve repair. In a rat model of acute SCI model, the combination strategy of topical application of FSZ NPs with GelMA hydrogel significantly improved motor function, reduced glial scarring, and promoted angiogenesis.

As a kind of MOF materials with high porosity and large surface area, ZIF-8 not only has good biocompatibility, but also can promote nerve repair [26]. Selenium is an antioxidant that plays vital roles in regulating redox and maintaining cell metabolism [51]. SeNPs could be controlled in size ranging from 2 to 200 nm by reducing selenite in an environment containing polyvinylpyrrolidone (PVP), which attaches to Se atoms and stops their aggregation [29]. According to the spatial arrangement of atoms, bimetallic nanocrystals can be divided into four categories: alloy or intermetallic compound, core-shell, core-satellite (or core-frame) and Janus nanocrystals [52–54]. In this study, three different sizes of SeNPs were successfully synthesized. These results suggest that different encapsulation strategies may be formed based on different sizes of nanocores.

It is well known that ROS overproduction during the acute to subacute phase of SCI leads to severe vascular system and neural network damage, and antioxidant therapy plays important roles in improving SCI prognosis [55, 56]. SeNPs are reported to be effective free radical scavengers that prevent ROS damage to neurons at lesion of SCI [57, 58]. Although SeNPs has less toxic and more biocompatible than organic or inorganic selenium compounds, it is still a cause for concern. Our results are similar to those previously reported, as the concentration of SeNPs of different sizes increases, PC12 cells immediately exhibit significant cytotoxicity. Compared with SeNPs, ZIF-8 can promote PC12 cell proliferation in a certain concentration range. This allows SeNPs to promote cell proliferation even after encapsulation, thus avoiding its inherent cytotoxicity. Because MIM-2 in ZIF-8 has certain ROS scavenging ability, SeNPs@ZIF-8 has better antioxidant function [23].

Nano-scale ZIF-8 and bimetallic complex materials has potential loading function, making it suitable for encapsulation of various cargos to develop nanocomposites with multiple biological functions [59]. Excessive ROS in central nervous system tissues can damage a variety of important biological components, such as nucleic acids, proteins, lipid acids, etc., further destroy the mitochondrial respiratory chain, release cytochrome C, induce caspase-3 activation and cause apoptosis of nerve cells [60, 61]. We selected Fer-1, a recognized iron death inhibitor, to further enhance the anti-neuronal death effect of SeNPs@ZIF-8. As expected, FSZ NPs demonstrated strong ROS clearance and completed mitochondrial protection both in vivo and in vitro, thereby alleviating neuronal apoptosis and ferroptosis. Macrophages play an important role in neuroinflammation. In the early stage, the classically activated type 1 (M1) macrophages phagocytose pathogens and damage cells and secrete pro-inflammatory factors; in the late stage, the alternatively activated type 2 (M2) macrophages secrete anti-inflammatory factors and devote to tissue repair and reconstruction [62]. However, excess ROS induces inflammatory cell infiltration and macrophage polarization through mitochondrial homeostasis disruption and production of pro-inflammatory factors, forming a vicious cycle of central nerve inflammation and ROS production, further aggravating inflammation and the formation of glial cell scar. In non-malignant conditions, selenium supplementation of macrophages attenuates the proinflammatory function of macrophages by switching macrophage activation from the pro-inflammatory phenotype M1 to the anti-inflammatory phenotype M2 [63, 64]. Our results suggest that FSZ NPs exhibit the ability to regulate macrophage polarization towards the protective phenotype M2. This may be due to the ability of FSZ NPs to inhibit the expression and release of inflammatory factors by regulating mitochondrial homeostasis. Mitochondria can influence the polarization state of macrophages by regulating metabolic pathways such as oxidative phosphorylation (OXPHOS) and aerobic glycolysis. In addition, hypoxia and free fatty acids affect mtROS production and mitochondrial dynamics, leading to mitochondrial dysfunction and mtDNA release, and promoting the M1 phenotype [65]. Mitochondrial disorders can directly or indirectly regulate the activation of various inflammatory bodies. Inhibition of OXPHOS and promotion of mtROS production increase NLRP3-dependent caspase-1 activity and promote the secretion of IL-1β and IL-18 [66]. Glial cells provide nutrients and protect neurons in the central nervous system while in an inflammatory state, they tend to form glial scars and act as a physical barrier to neuronal regeneration [67]. When spinal cord injury occurs, endogenous neural stem cells (NSCs) are affected by inflammatory factors secreted by abnormal macrophage polarization, and astrocyte differentiation occurs instead of neurons [68]. FSZ NPs inhibited the occurrence of glial scar by successfully controlling the polarization of macrophages and regulating the expression of inflammatory factors.

Sustained zinc ion release significantly promotes neural differentiation of stem cells, but the molecular mechanism is not fully understood [26, 69]. Similar to our findings, FSZ NPs significantly promoted the neural differentiation and maturation of PC12 cells. Previous studies indicated that WNT4 significantly promotes axon growth and neuronal maturation of neural stem cells by activating both canonical (β-catenin dependent) and non-canonical WNT (JNK/P38 dependent) signaling pathways [70]. Both canonical and non-canonical activation of WNT signaling can induce axon regeneration and neurite growth, suggesting that exogenous stimulation pathways can reassert the role of WNT after adult SCI injury [71, 72]. Our results showed that with the increase of FSZ NPs concentration, various neural differentiation indexes increased gradually. In addition, FSZ NPs activation of β-catenin signaling pathway depends on WNT4 expression. This suggests that WNT4 is very important for the neuro-promoting function of FSZ NPs.

The early construction of blood vessels at SCI injury is very important for regulating the local hypoxia and nutrient deprivation microenvironment, thus inhibiting the apoptosis of neurons [73]. There is a direct relationship between the breakdown of the blood-spinal barrier (BSCB) after spinal cord injury and damage to the cell types that form the spinal cord [74]. After SCI, mitochondrial ROS levels in vascular endothelial cells are elevated due to increased oxygen consumption, which affects the integrity of BSCB. Therefore, promoting angiogenesis and maintaining BSCB stability is essential for maintaining mitochondrial homeostasis to control high levels of ROS after spinal cord injury. FSZ NPs also has a significant function of promoting angiogenesis in vivo and in vitro, which can be attributed to its significant promotion of VEGF expression.

## Conclusion

Here, multiple synthetic strategies based on SeNPs have been developed that significantly improves the therapeutic window of nano-selenium, which allows non-toxic doses of FSZ NPs to obtain excellent ROS scavenging effect to blocks the death of neurons from ferroptosis and apoptosis. Through ROS scavenging of mitochondria, FSZ NPs maintain mitochondrial homeostasis, thus regulates the release of inflammatory cytokines and the polarization of macrophages. In addition, FSZ NPs can promote axon growth and angiogenesis by releasing Zn^2+^ to active the WNT/β-catenin signaling pathway. In summary, this study developed a series of novel SeNPs-based nanocomposites, which provides an effective and promising anti-oxidant therapeutic strategy for the treatment of SCI (Scheme 1).

### Electronic supplementary material

Below is the link to the electronic supplementary material.

Supplementary Material 1

## Data Availability

No datasets were generated or analysed during the current study.

## Abbreviations

2-MIM: 2-methylimidazole
TSQ: N- (6-Methoxy-8-quinolyl)-p-toluenesulfonamide
ABTS: 2,2′-Azinobis-(3-ethylbenzthiazoline-6-sulphonate)
BAX: BCL-2-associated X, apoptosis regulator
BSCB: Blood-spinal cord barrier
CHL: Chloroquine
CPZ: Chlorpromazine
DMEM: Dulbecco’s modified eagle medium
ECM: Endothelial cell medium
FBS: Fetal bovine serum
Fer-1: Ferrostatin 1
FSZ: Fer-1@SeNPs@ZIF-8
GSSG: Glutathione oxide
H&E: Hematoxylin and Eosin
HS: Horse Serum
HUVECs: Human Umbilical Vein Endothelial Cells
IL-6: Interleukin 6
MβCD: Methyl-β-cyclodextrin
MSCs: Mesenchymal stem cells
mtDNA: Mitochondrial DNA
PBS: Phosphate-buffered saline
PVDF: Polyvinylidene fluoride
PVP: Polyvinylpyrrolidone
RIPA: Radioimmunoprecipitation assay
ROS: Reactive oxygen species
RPMI-1640: Roswell Park Memorial Institute 1640 medium
SCI: Spinal cord injury
SeNPs: Selenium nanoparticles
SSe: Small Selenium
SSSe: Small-small Selenium
TBHP: Tert-Butyl hydroperoxide
TEM: Transmission electron microscopy
TMB: 3,3,5,5-tetramethylbenzidine
TNF: Tumor necrosis factor
XRD: X-ray diffraction
ZIF-8: Zeolitic imidazolate framework-8
