# Supplementary material for: Repair spinal cord injury with a versatile anti-oxidant and neural regenerative nanoplatform
Source: J Nanobiotechnology. 2024 Jun 20;22:351. doi: 10.1186/s12951-024-02610-5 (PMC11188197; doi:10.1186/s12951-024-02610-5)
Supplement: Supplementary file 1 — Supplementary Material 1 [file 12951_2024_2610_MOESM1_ESM.docx]

**Repair spinal cord injury with a versatile anti-oxidant and neural regenerative nanoplatform**

Heng Zhou^1#^, Ziwei Li^1#^, Shuili Jing^1#^, Ben Wang^2^, Zhifei Ye^2^, Wei Xiong^1^, Yonghao Liu^1^, Ye Liu^1^, Chun Xu^3^, Tushar Kumeria^4^, Yan He^5,6^, Qingsong Ye^1,2*^

1 Center of Regenerative Medicine & Department of Stomatology, Renmin Hospital of Wuhan University, Wuhan, 430060, China

2 The Second People’s Hospital of Linhai, Linhai, Zhejiang, 317000, China

3 School of Dentistry, The University of Sydney, Sydney, NSW, 2006, Australia

4 School of Materials Science and Engineering, University of New South Wales, Kensington, Sydney, NSW, 2052, Australia

5 Institute of Regenerative and Translational Medicine, Tianyou Hospital, Wuhan University of Science and Technology, Wuhan, Hubei, 430064, China

6 Department of Oral and Maxillofacial Surgery, Massachusetts General Hospital, Harvard Medical School, Boston, 02114, MA, USA

*Corresponding authors:

Qingsong Ye

Center of Regenerative Medicine, Renmin Hospital of Wuhan University, Wuhan, China. Email: [qingsongye@whu.edu.cn](mailto:qingsongye@whu.edu.cn)

#These authors contributed equally to this work.

**
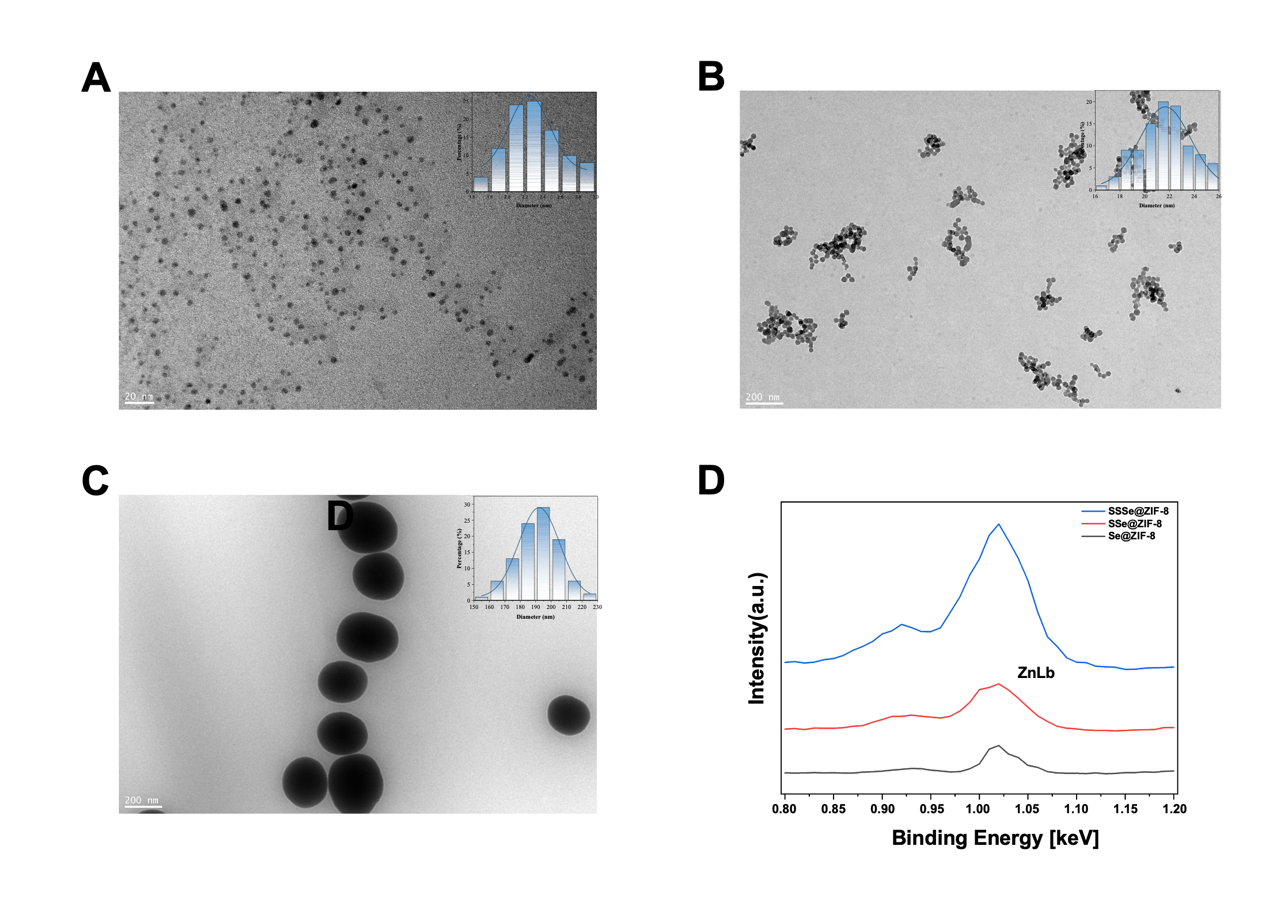
Supplementary Data**

**Figure S1. Identification of Se, SSe, and SSSe NPs.**

A-C) TEM images and Size distribution of SSSe, SSe, and Se nanoparticles. D) energy spectrum analysis of ZnLb spectra of SSSe@ZIF-8, SSe@ZIF-8, and Se@ZIF-8.


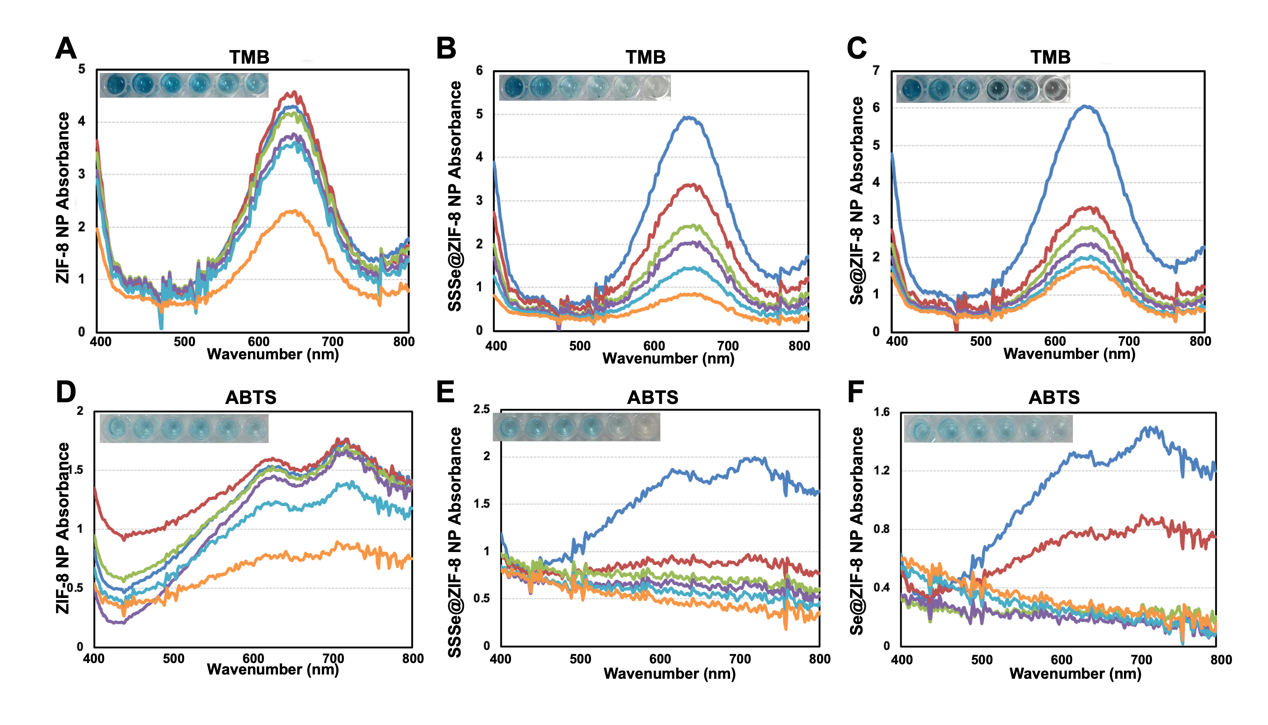


**Figure S2. ROS scavenging of ZIF-8, SSSe@ZIF-8 and Se@ZIF-8 *in vitro*.**

(A-C) The concentration-dependent anti-oxidation of TMB due to •OH elimination by ZIF-8, SSSe@ZIF-8, and Se@ZIF-8 with 100 μM H2O2. (D-F) The concentration-dependent investigation of ABTS•+ in the presence of ZIF-8, SSSe@ZIF-8, and Se@ZIF-8.

**
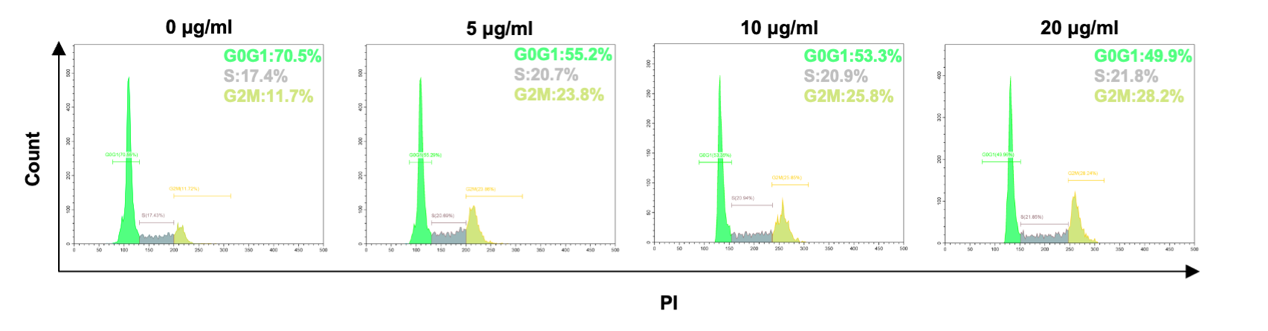
****Figure S3. Cell cycle analysis of FSZ nanoparticles on PC12 cells.** Flow cytometry analysis of cell cycle changes in PC12 cells treated with different concentration of FSZ nanoparticles.

**
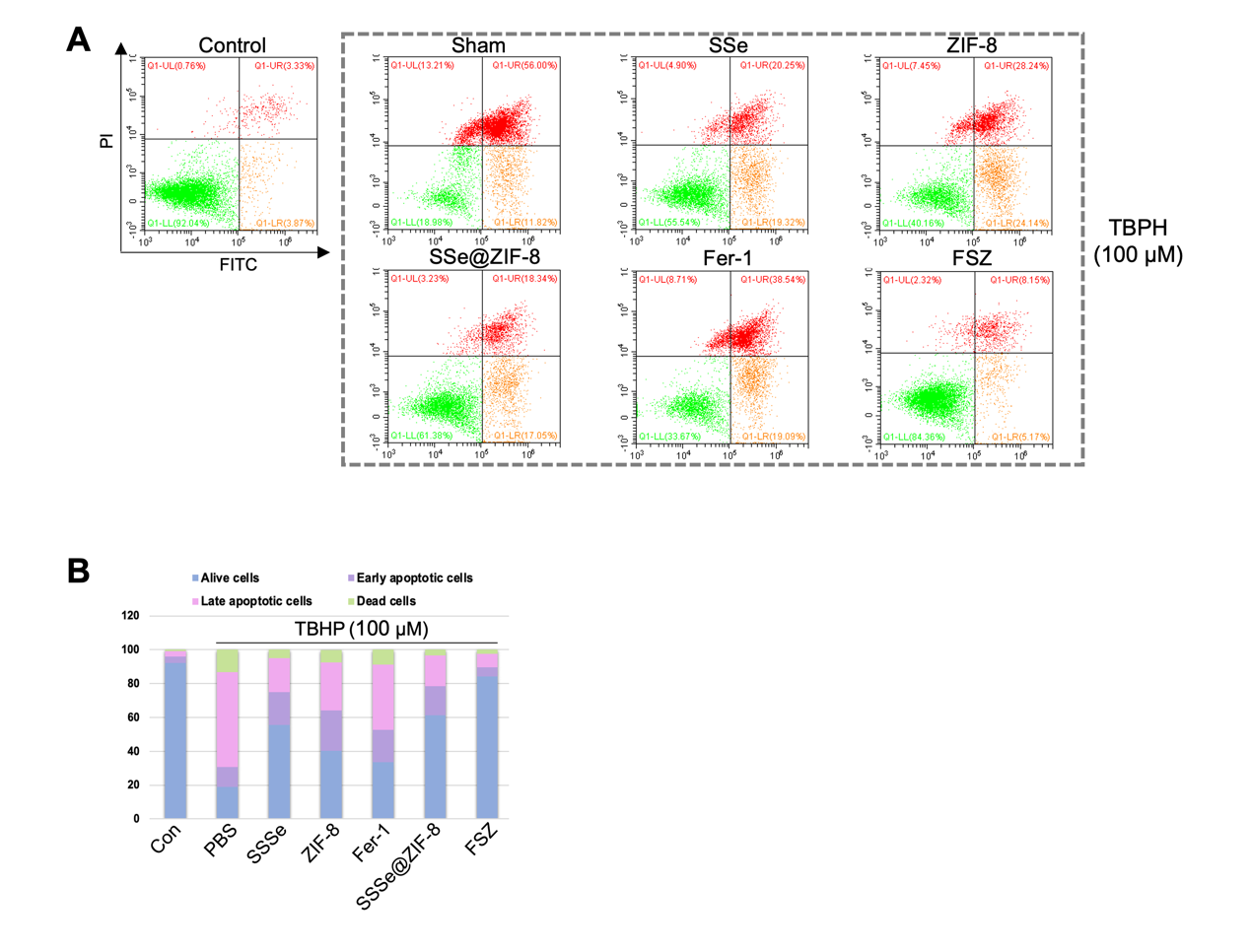
**

**Figure S4. FSZ nanoparticles inhibit apoptosis induced by ROS accumulation.**

PC12 cells were co-cultured using 100 μM TBHP and PBS, SSSe, ZIF-8, Fer-1, SSe@ZIF-8, and FSZ nanoparticles. (A) Flow cytometry analysis of annexin‐V/PI staining in PC12 cells treated with TBHP (100 μM). (B) The distribution of apoptosis was quantified.

**
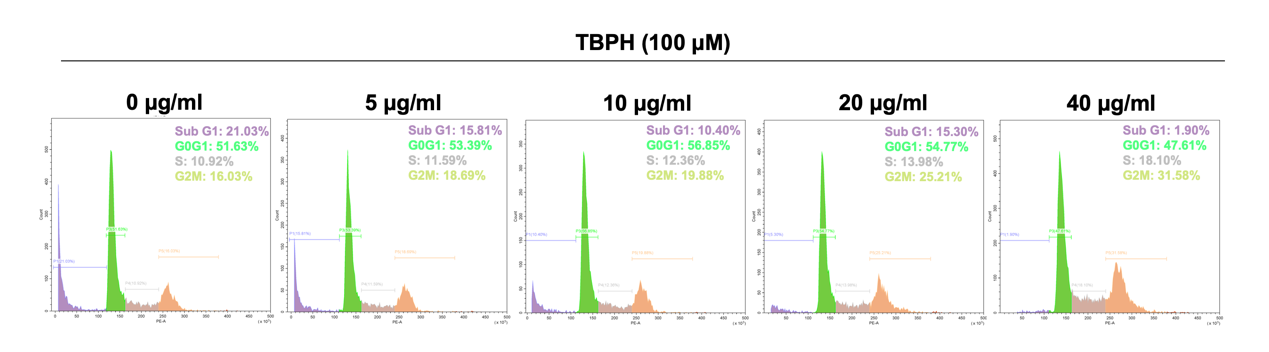
**

**Figure S5. Cell cycle analysis of FSZ nanoparticles on PC12 cells treated with ROS induction.** Flow cytometry analysis of cell cycle changes in PC12 cells treated with different concentration of FSZ nanoparticles with in TBHP (100 μM).

**
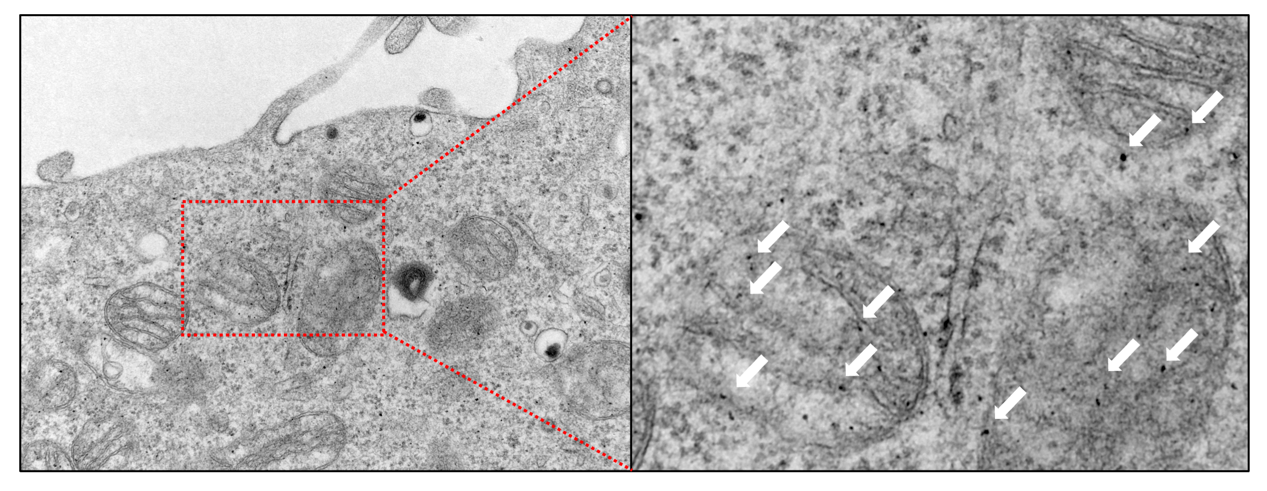
**

**Figure S6. FSZ nanoparticles target mitochondria.** PC12 cells were treated with FSZ nanoparticles (100 μg/ml) and TBHP (100 μM). The organelle state and nanoparticle distribution were observed by transmission electron microscopy.

**
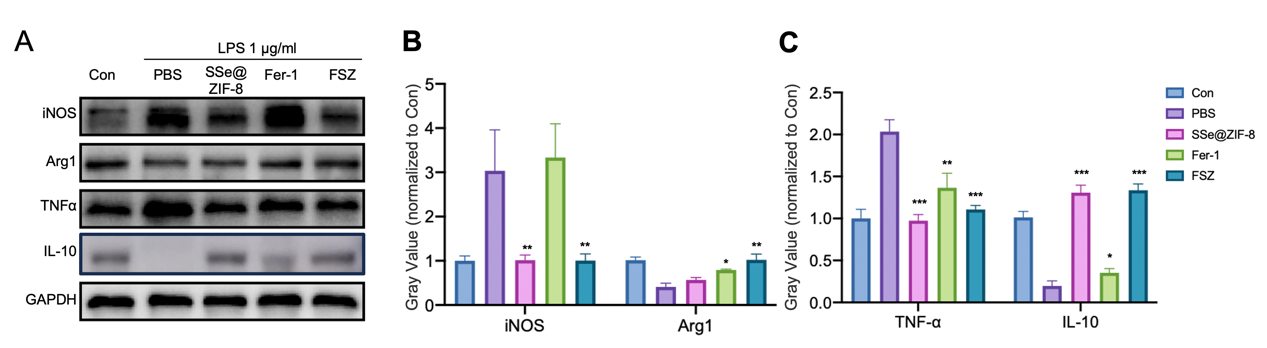
**

**Figure S7. FSZ nanoparticles affect macrophage polarization.** Raw264.7 cells were incubated with 20 μg/ml SSe@ZIF-8, Fer-1 (1 μM) and FSZ combination with 1 μg/ml LPS for 24 h. (A-C) Protein levels of iNOS, Arg1, TNFα and IL-10 were detected by Western blot. Data are presented as means ± SD (n = 3). Statistical analysis was performed using one-way ANOVA. *P < 0.05, **P<0.01, ***P < 0.001.

**
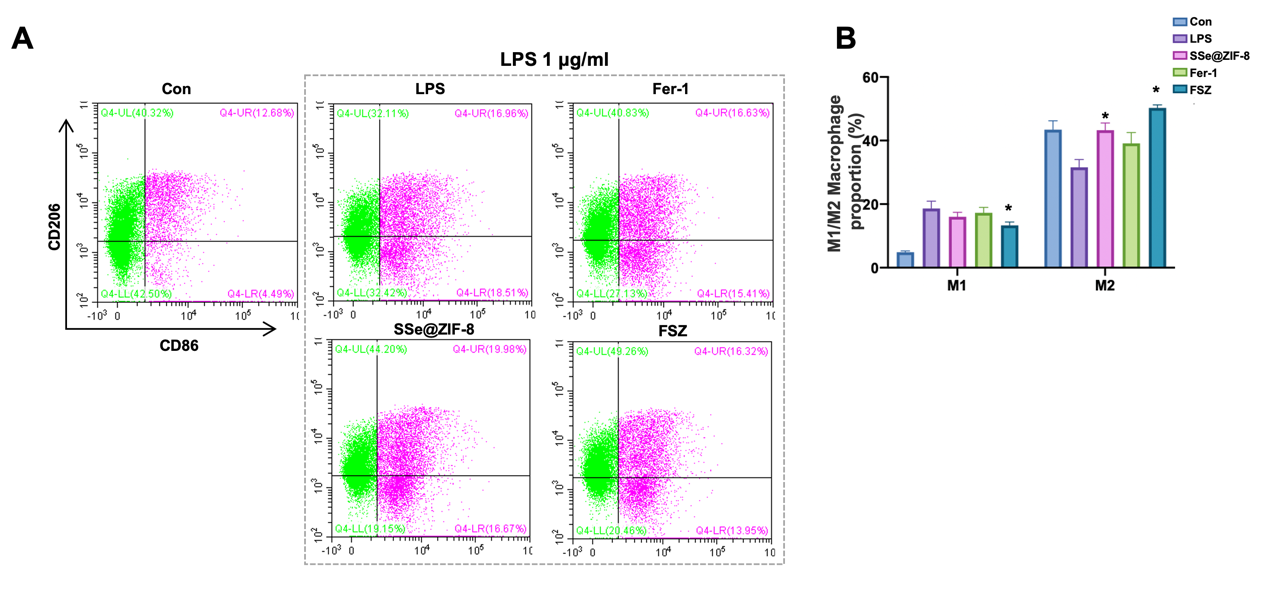
**

**Figure S8. FSZ nanoparticles promote the differentiation of macrophages to M2 phenotype.** Raw264.7 cells were incubated with 20 μg/ml SSe@ZIF-8, Fer-1 (1 μM) and FSZ combination with 1 μg/ml LPS for 24 h. The flow cytometry analysis Raw264.7 cells (CD206 and CD86) was shown. Statistical analysis was performed using one-way ANOVA. *P < 0.05.

**
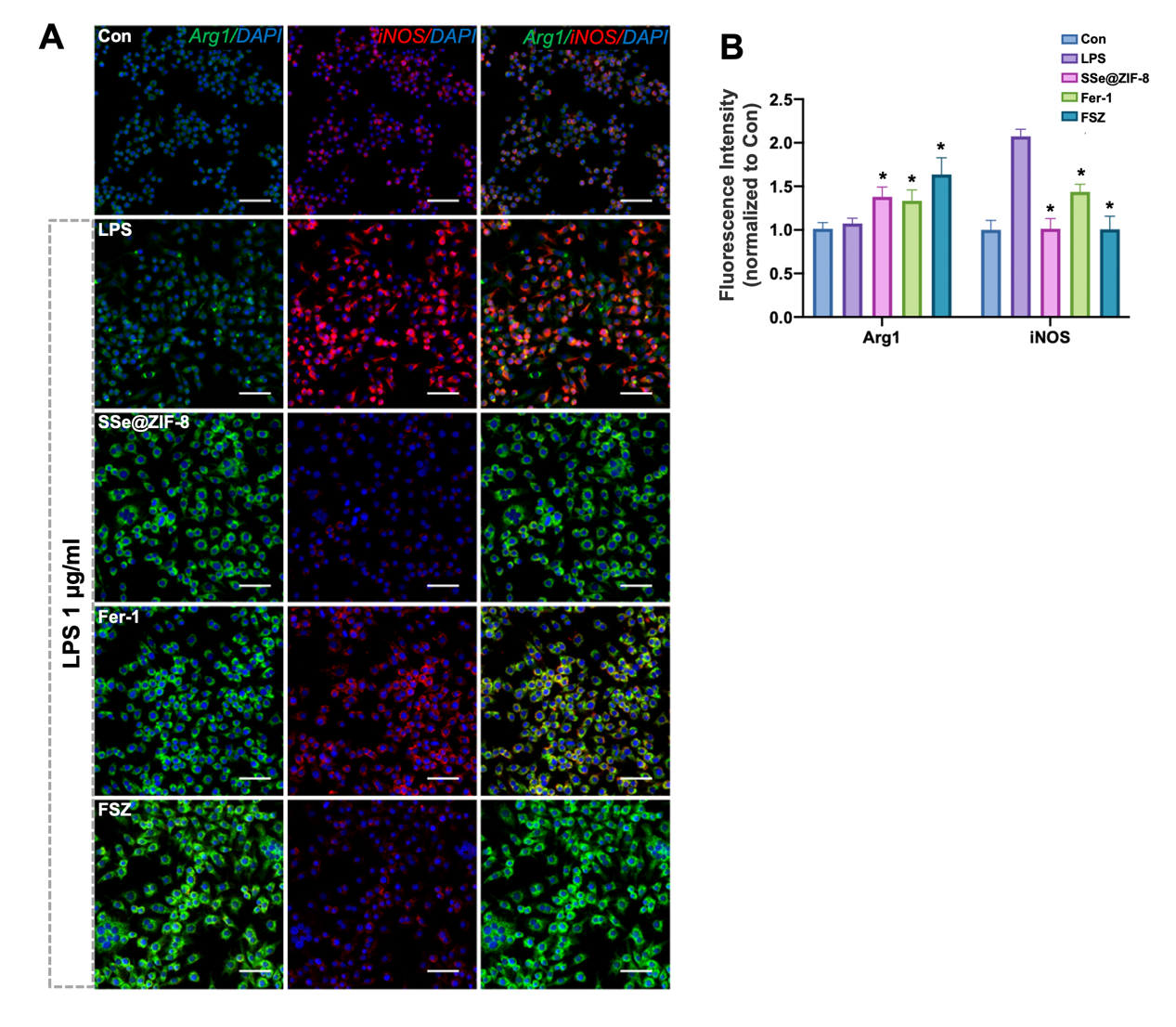
**

**Figure S9. Immunofluorescence analysis of FSZ nanoparticles on macrophage polarization.** Raw264.7 cells were incubated with 20 μg/ml SSe@ZIF-8, Fer-1 (1 μM) and FSZ combination with 1 μg/ml LPS for 24 h. (A) Representative pictures of fluorescence analysis of iNOS and Arg1 were given. (B) Quantitative analysis of iNOS and Arg1 expression. Statistical analysis was performed using one-way ANOVA. *P < 0.05.

**
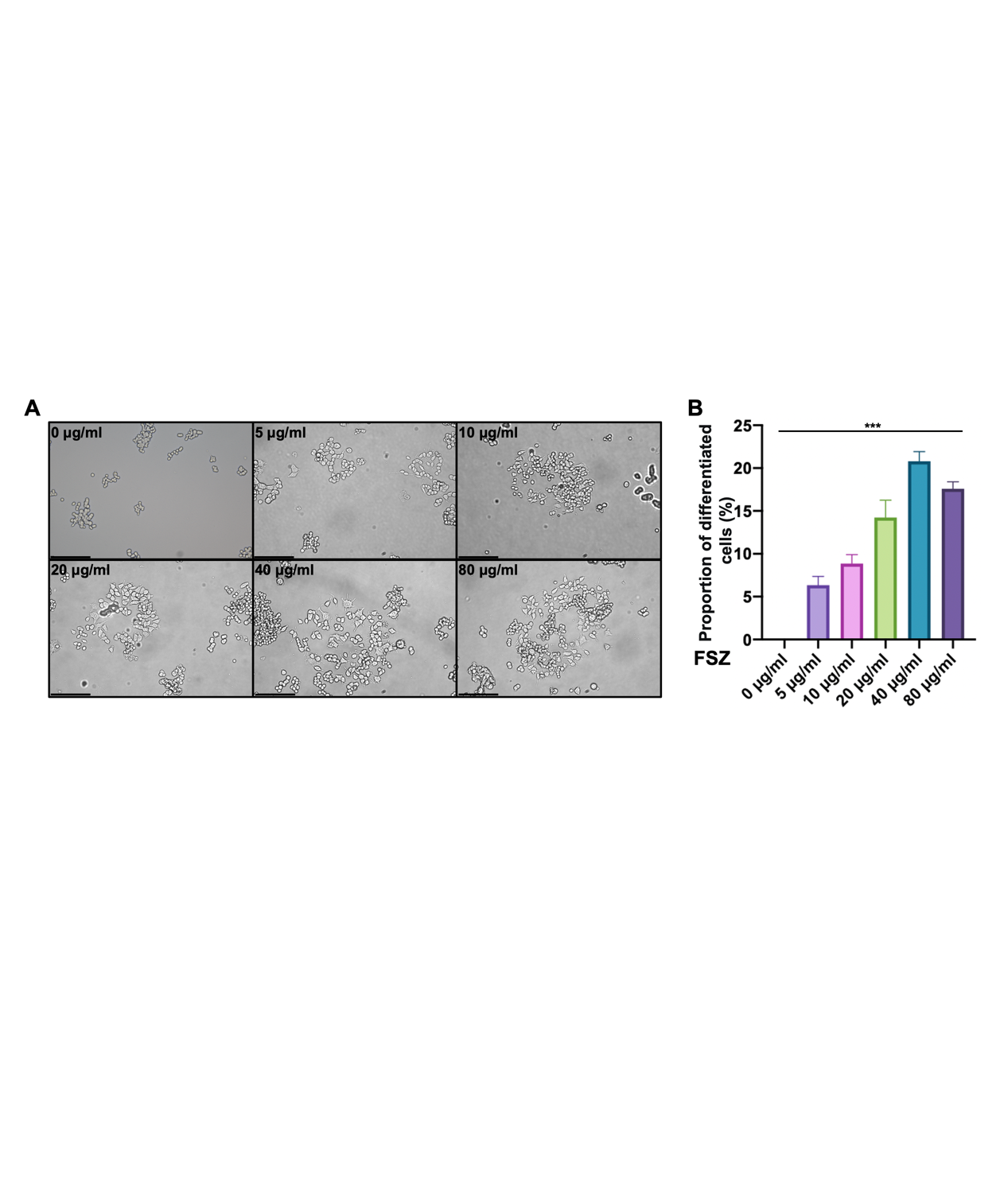
**

**Figure S10. FSZ nanoparticles contribute the autonomous differentiation of PC12 cells.**

(A) Bright field images of PC12 cells incubated with FSZ for 6 days in ordinary medium. Scale bar: 100 μm. (B) The different ratio of PC12 cells incubated with FSZ for 6 days in ordinary medium. Data are presented as means ± SD (n = 3). Statistical analysis was performed using one-way ANOVA. ***P < 0.001.

**
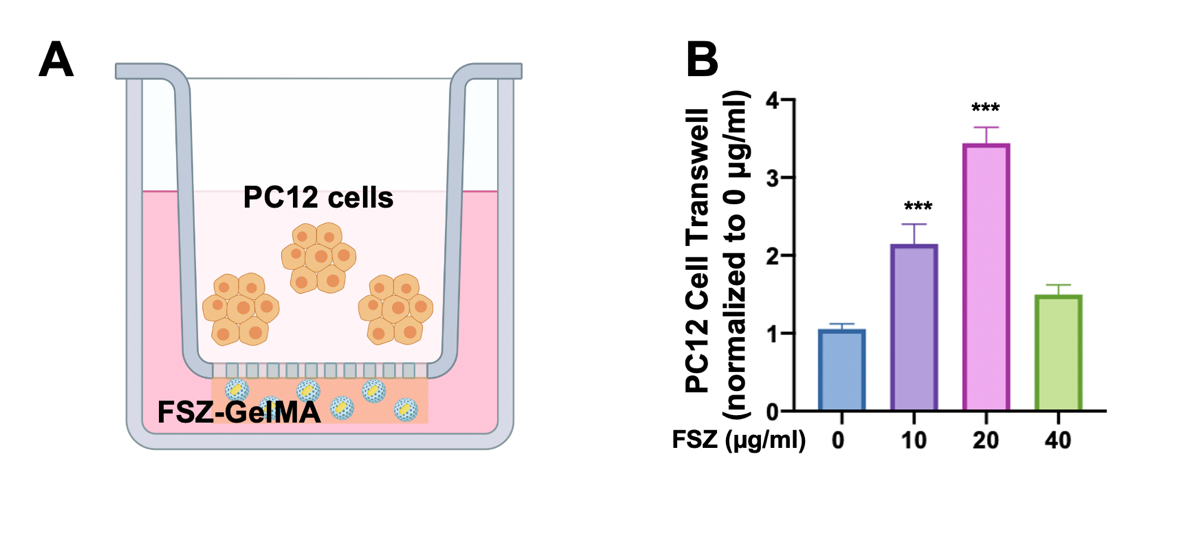
**

**Figure S11. FSZ promotes PC12 cell recruitment.** (A) Schematic illustration of the co-culture of PC12 cells and FSZ-GelMA. (B) The transport ratios of PC12 cells from apical chamber to FSZ-GelMA after 24 h was determined by CCK-8 assay. Statistical analysis was performed using one-way ANOVA. ***P < 0.001.

**
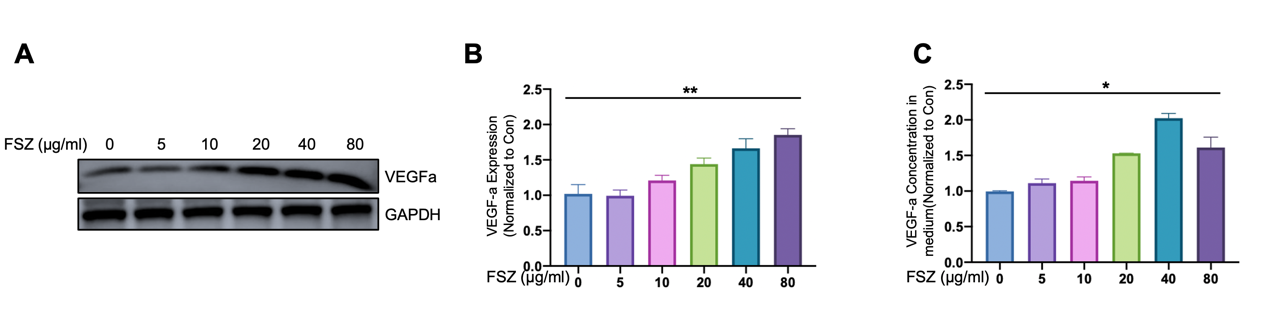
**

**Figure S12. FSZ promotes VEGFa expression.** PC12 cells were incubated with different concentrations of FSZ (0, 5, 10, 20, 40 and 80 μg/ml) for 24 h. (A) Western blot was used to detect expression of VEGFa and GAPDH. (B) VEGFa expression was quantified. (C) ELISA analysis of VEGF-a of the supernatant from HUVECs incubated with different concentrations of FSZ for 24 h. Data are presented as means ± SD (n = 3). Statistical analysis was performed using one-way ANOVA. *P < 0.05, **P<0.01.

**
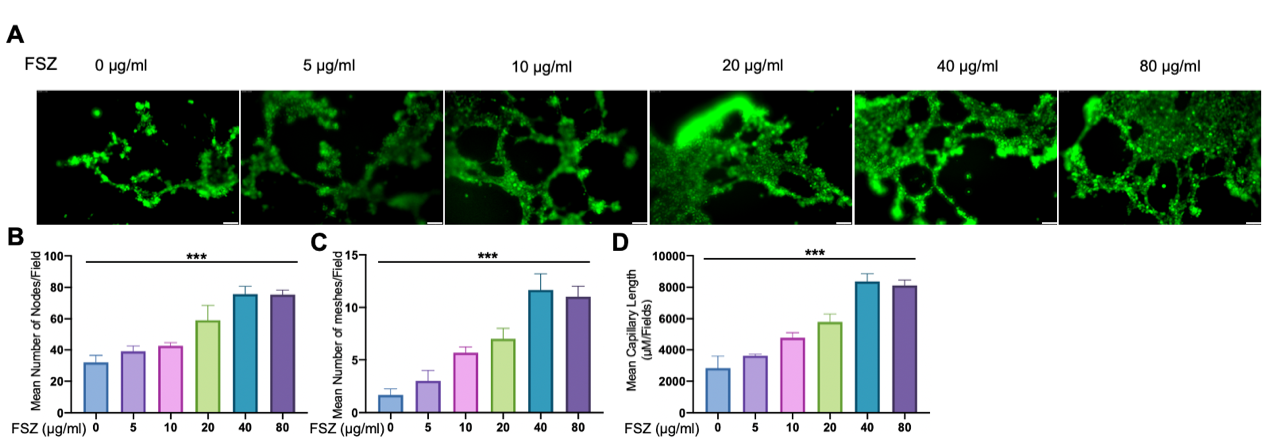
**

**Figure S13. FSZ promotes blood vessel formation.** PC12 cells were incubated with different concentrations of FSZ (0, 5, 10, 20, 40 and 80 μg/ml) for 24 h. The conditioned medium was collected to treated with HUVECs to perform tube formation assay for 6 h. Representative images of CalceinAM-labeled HUVECswas given. (B-D) Quantitative analysis of the number of nodes, the number of meshes, and tube length based of tube formation assay. Data are presented as means ± SD (n = 3). Statistical analysis was performed using one-way ANOVA. ***P<0.001.


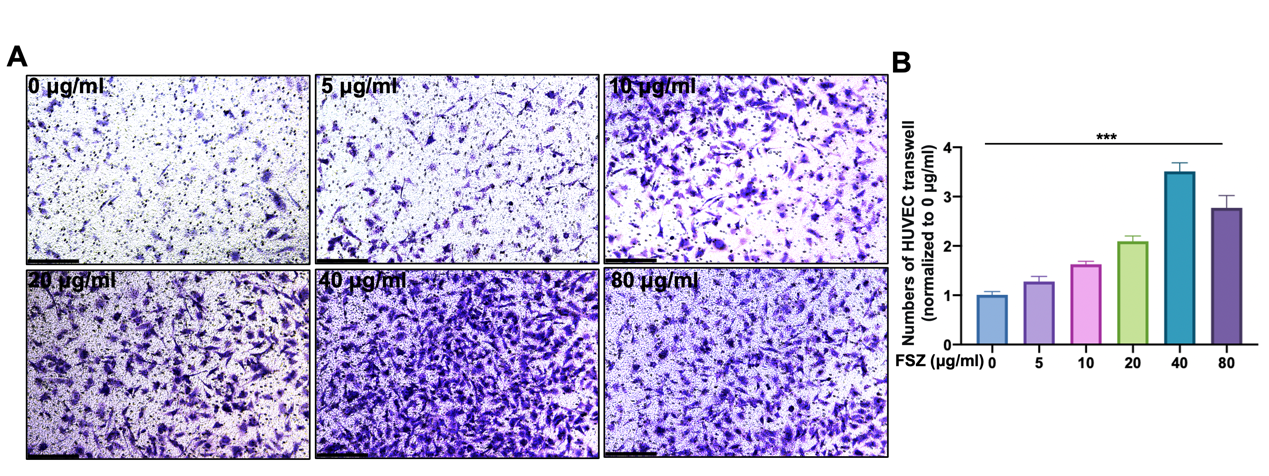


**Figure S14. FSZ promotes migration of HUVECs.** PC12 cells were treated with different concentrations of FSZ (0, 5, 10, 20, 40 and 80 μg/ml) and co-cultured with HUVECs for 24 h. (A) Representative images of trans-well migration assay of HUVECs. Scale bar: 100 μm. (B) Quantitative analysis of trans-well migration assay Data are presented as means ± SD (n = 3). Statistical analysis was performed using one-way ANOVA. ***P < 0.001.

**
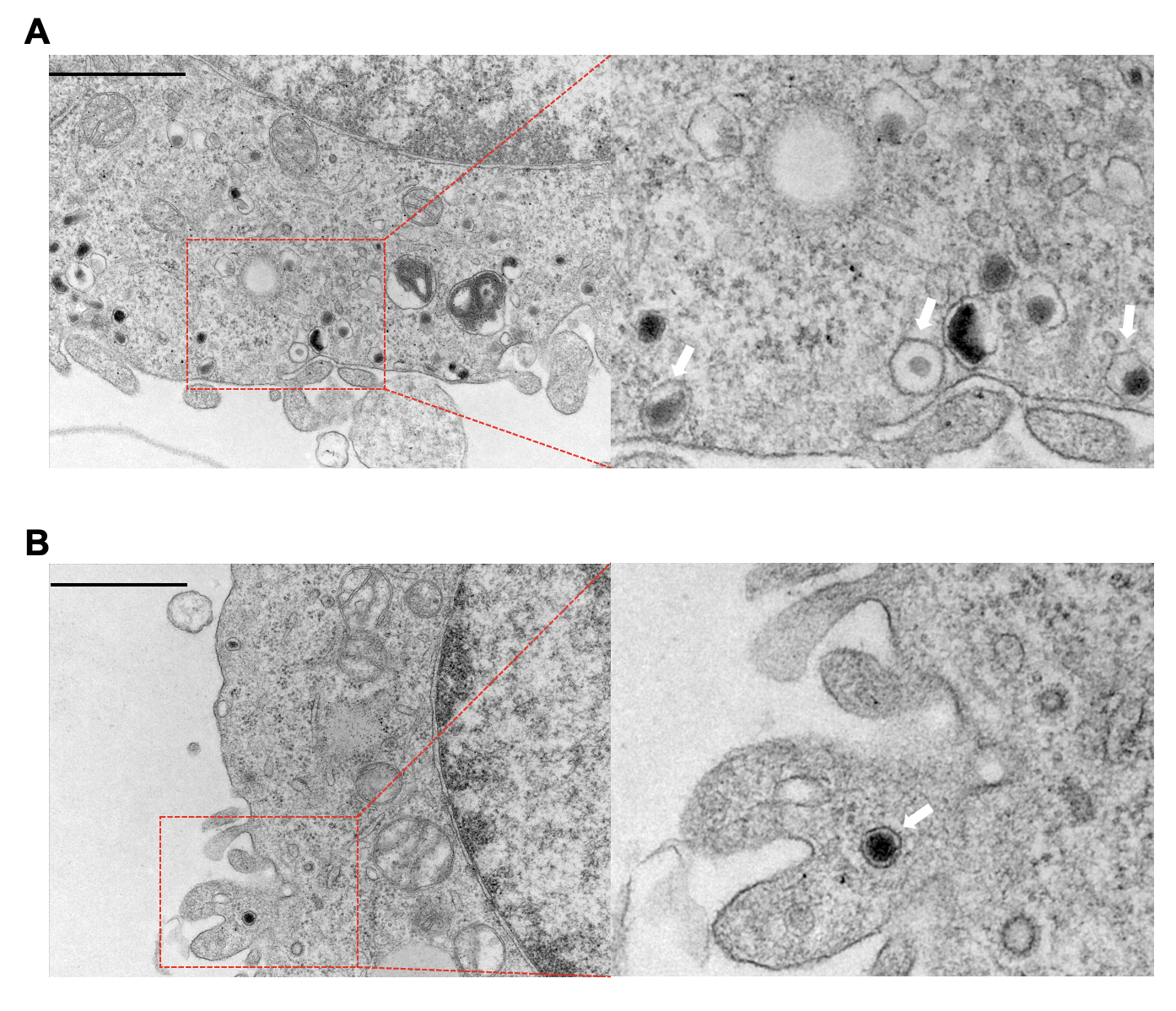
**

**Figure S15. The endocytosis mechanism of SSe@ZIF-8 nanoparticles**

A) TEM images of endocytosis of PC12 cells treated with SSe@ZIF-8. B) TEM images of macropinocytosis cups of PC12 cells treated with SSe@ZIF-8. Scale bar: 1 μm. white arrow: SSe@ZIF-8 in lysosomes.

**
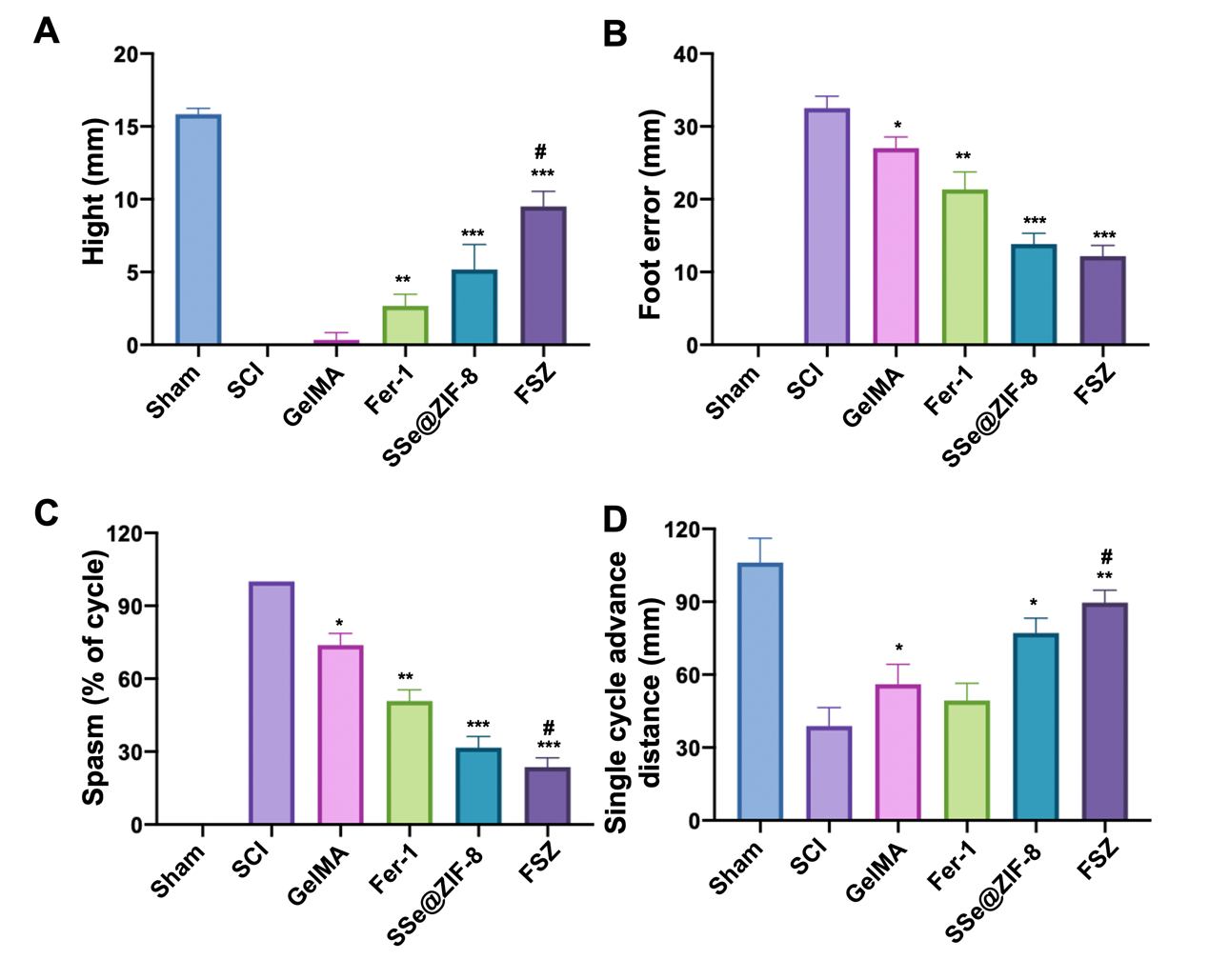
**

**Figure S16. FSZ nanoparticles promote the recovery of motor function in SCI rats**

A-D) height from the ground, foot error, spasm duration and forward distance of the motion of mice in the video. Data are presented as means ± SD (n = 3). Statistical analysis was performed using t-test. # P < 0.05, compared with SSe@ZIF-8 group; * P < 0.05, ** P < 0.01, and *** P < 0.001 compared with SCI group.

**
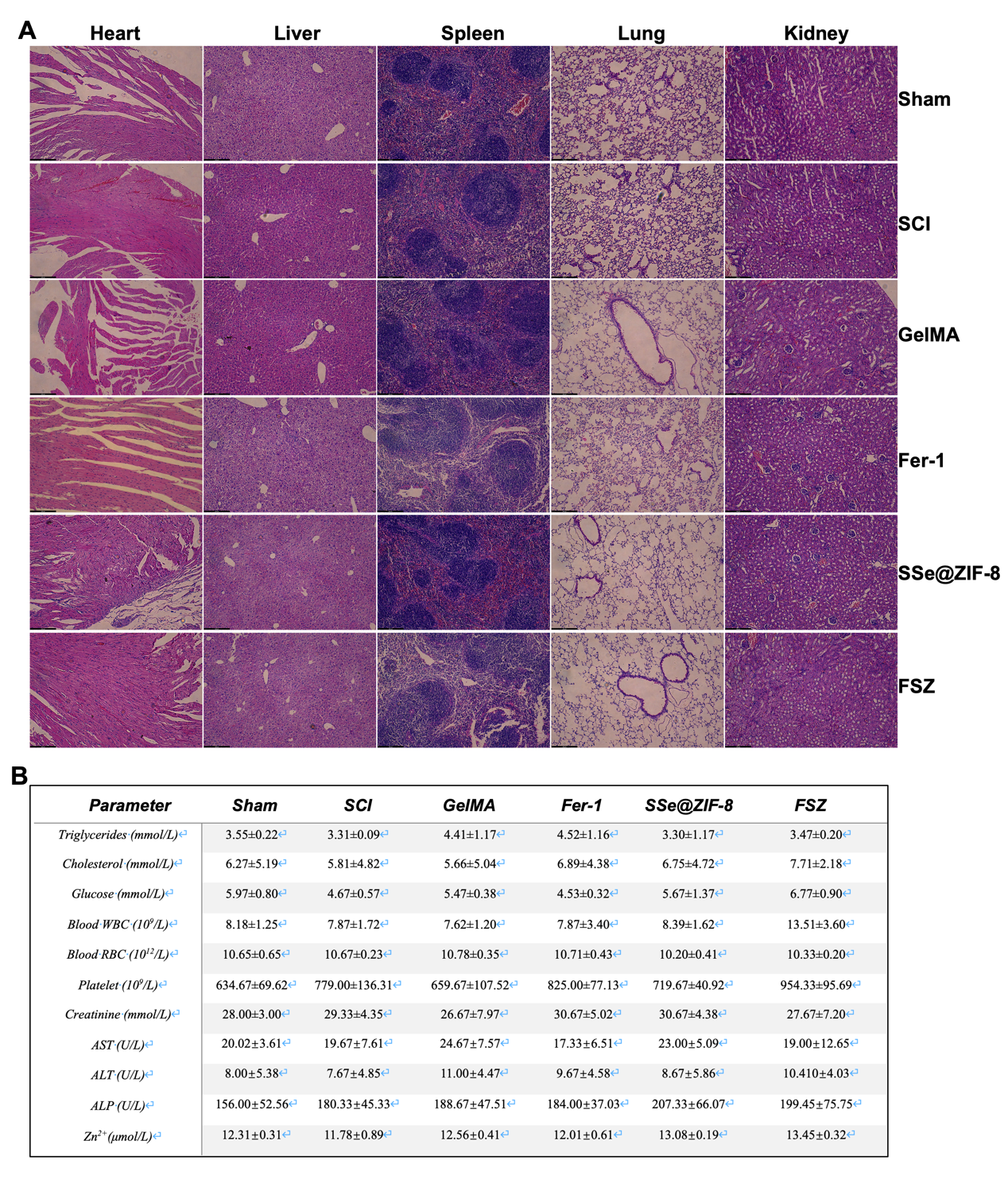
Figure S17. Safety assessment of FSZ nanoparticles in vivo**

A) H&E staining image of heart, liver, spleen, lung, and kidney of mice. B) Blood routine examination of the anticoagulant tail blood of mice.

**
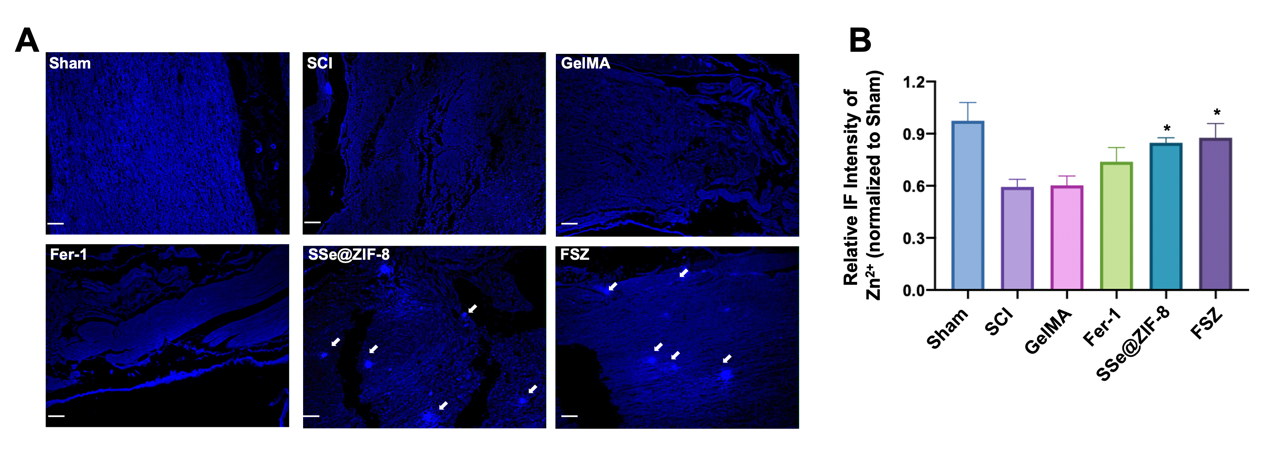
**

**Figure S18. FSZ nanoparticles promote Zn^2+^ levels in spinal cord injury.** (A-B) Quantitative analysis of the content of Zn^2+^ by TSQ staining. White arrow: high concentration of Zn^2+^. Data are presented as means ± SD (n = 3). * P < 0.05 compared with SCI group.


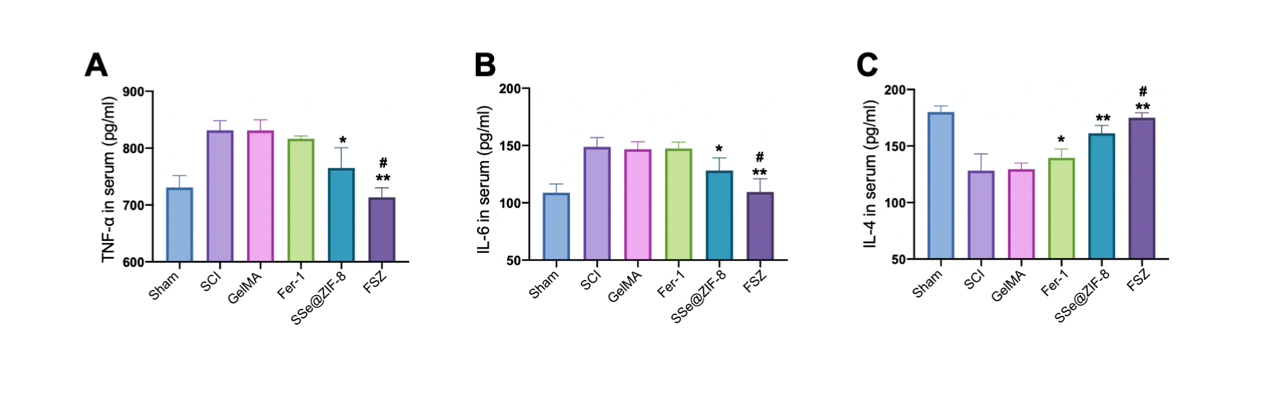


**Figure S19. FSZ nanoparticles regulate the release of inflammatory factors in serum.** (A-C) ELISA assay was used to detect TNF-α, IL-6 and IL-4 levels in serum. Data are presented as means ± SD (n = 3). * P < 0.05 compared with SCI group.


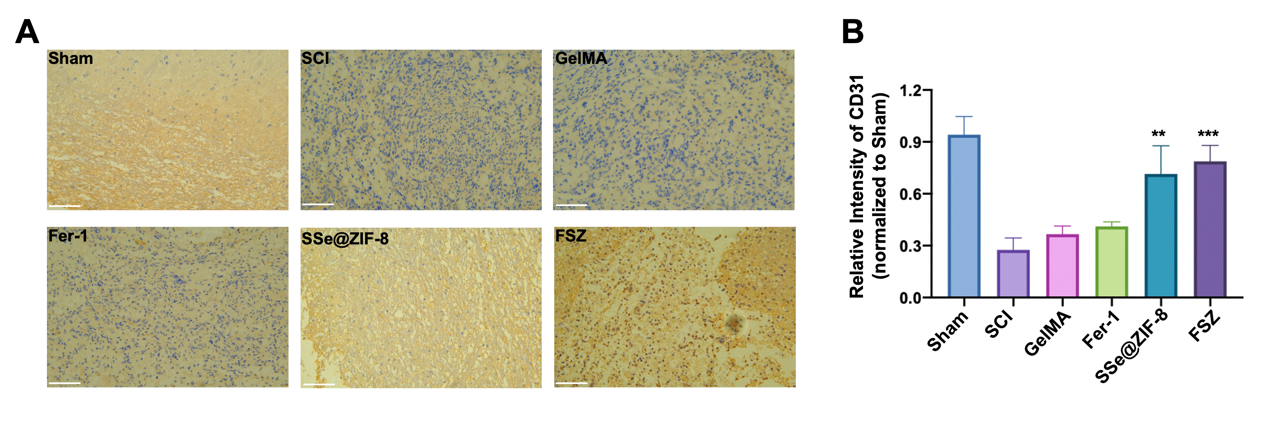


**Figure S20. FSZ nanoparticles promote angiogenesis *in vivo***. (A) Immunohistochemical images of CD31 indicating angiogenesis. Scale bar: 200 μm. B) Quantitative analysis of the expression of CD31. Data are presented as means ± SD (n = 3). Statistical analysis was performed using t-test. ** P < 0.01, and *** P < 0.001 compared with SCI group.

**Supplementary Table 1. The sequences of siRNAs used in the study**

| si-RNA Sence(5′-3′) antisence(5′-3′) |
| --- |
| si-WNT4-1 UCCACACUCGACUCCUUGCTT GCAAGGAGUCGAGUGUGGAGC  si-WNT4-2 AGGAGACGUGCGAGAAACUTT AGUUUCUCGCACGUCUCCUCC  control UUCUCCGAACGUGUCACGUTT ACGUGACACGUUCGGAGAATT |
